# Mg_44.29_Ru_7_

**DOI:** 10.1107/S2414314625007291

**Published:** 2025-08-19

**Authors:** Junhui Li, Changzeng Fan, Bin Wen, Lifeng Zhang

**Affiliations:** a State Key Laboratory of Metastable Materials Science and Technology Yanshan University,Qinhuangdao 066004 People’s Republic of China; b Hebei Key Lab for Optimizing Metal Product Technology and Performance Yanshan University,Qinhuangdao 066004 People’s Republic of China; c School of Mechanical and Materials Engineering North China University of Technology,Beijing 100144 People’s Republic of China; Vienna University of Technology, Austria

**Keywords:** crystal structure, high-pressure synthesis, inter­metallics, Mg–Ru_7_ phase

## Abstract

A cubic phase with composition Mg_44.29_Ru_7_ (tetra­tetra­contamagnesium hepta­ruthenium) was obtained during high-pressure sinter­ing of a mixture with an initial chemical composition of MgRuB. Mg_44.29_Ru_7_ has space-group symmetry *F*43*m* and adopts the Mg_29 – *x*_Pt_4 + *y*_ type of structure, which is categorized as one of the two structural types identified in complex *A*_6_*B* compounds.

## Abstract 2

Cubic Mg_44.29_Ru_7_ is related to the previously reported Mg_43.83_Ru_7.17_ to which it shows differences relative to some occupancies of the Ru and Mg sites,

## Graphical abstract

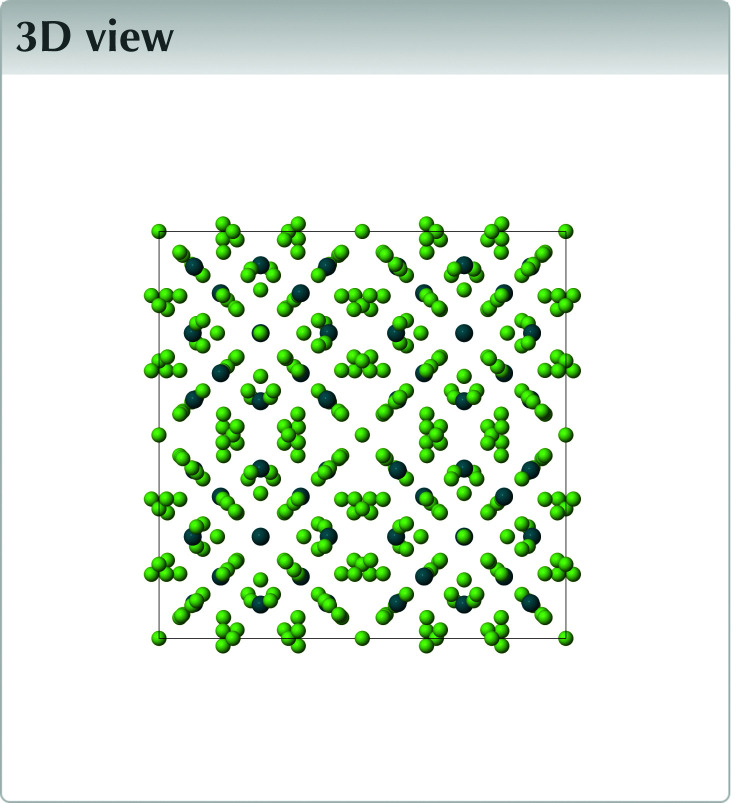

## Structure description

There are two structural types in complex compounds with idealized formula *A*_6_*B* crystallizing in the cubic space group *F*
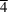
3*m*. For example, the Mg_44_Rh_7_ phase discovered by Westin (1971 ▸) belongs to the first structure type with lattice parameter *a* = 20.148 Å. Westin & Edshammar (1972 ▸) reported on a related magnesium-rich compound in the Mg/Ir system, Mg_44_Ir_7_, which is isotypic with Mg_44_Rh_7_. Based on Westin’s work (Westin, 1972 ▸), Andersson (1978 ▸) pointed out that Mg/Ru phases near the composition Mg_44_Rh_7_ have a large homogeneity range so that ‘slightly different phases appear upon heat treatment at relatively low temperatures of Mg_44_Ru_7_^’^. Such a different cubic phase with composition of Mg_44.29_Ru_7_ was obtained in the present study during high-pressure sinter­ing of a mixture with an initial chemical composition of MgRuB. The lattice parameter *a* is similar to the aforementioned Mg_44_Rh_7_ phases. The refined composition of the Mg_44.29_Ru_7_ phase aligns closely with the elemental ratios determined by energy-dispersive X-ray spectroscopy (EDX) analysis (see Table S1 of the supporting information).

**Figure 1:**
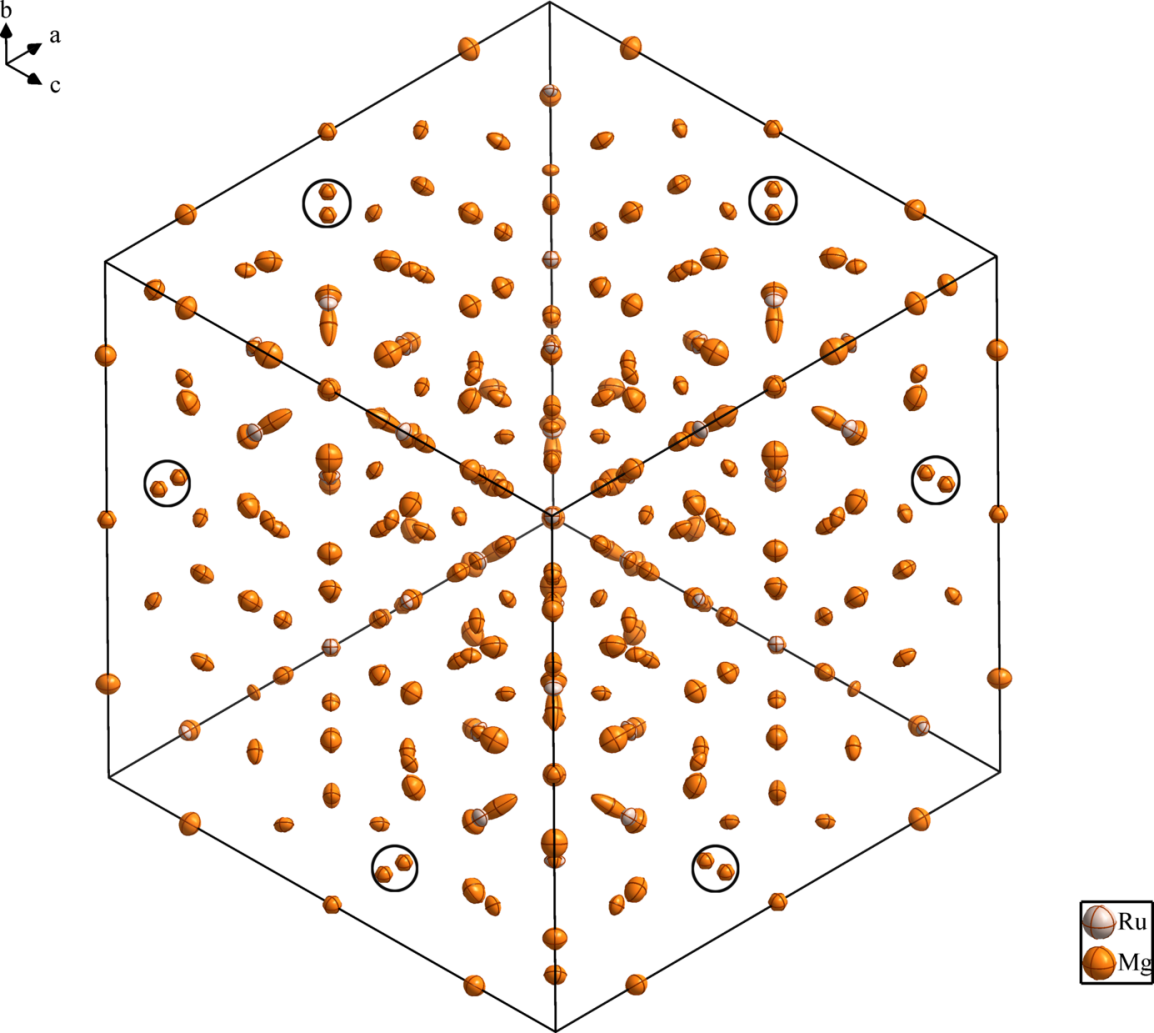
The crystal structure of Mg_44.29_Ru_7_ (showing one unit cell projected along [111]), with displacement ellipsoids drawn at the 99% probability level. The circles represent partially split Mg11 and Mg13 sites.

The present Mg_44.29_Ru_7_ phase (Fig. 1 ▸) belongs to the second structure type of complex *A*_6_*B* compounds, just like the two phases in the Mg/Pd and Mg/Pt systems. Samson (1972 ▸) worked out that the basic building blocks of the Mg_340.04_Pd_55.84_ phase consist of eight icosa­hedra, four tri-capped trigonal prisms and 48 penta­gonal prisms. Four such building blocks share a Laves–Friauf polyhedron, the centre of which is occupied in this case by the Mg11 atomic position (multiplicity 4, Wyckoff letter *d*), but is empty in Mg_44_Rh_7_ (first type). Agnarelli *et al.* (2022 ▸) performed chemical bonding and structure analyses of Mg_29 – *x*_Pt_4 + *y*_ phases, revealing that the same central position is partially occupied by Mg8 atoms (multiplicity 4, Wyckoff letter *a*), and the nearest Mg position is split into Mg11 (when Mg8 is present) and Mg12 (when Mg8 is unoccupied). In the case of Mg11, this leads to a rather unusual partial occupancy by Pt. The final refinement resulted in occupancies of 58% Mg + 5% Pt for the Mg11 site and 15% Mg for the Mg12 site. During the refinement of the crystal structure of Mg_44.29_Ru_7_, it was confirmed that it exhibits a similar atomic environment at the same central position as the Mg_29 – *x*_Pt_4 + *y*_ phases. The central position is characterized by partial occupancy by Mg1 (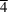
3*m* symmetry, multiplicity 4, Wyckoff letter *b*), and is further subdivided into Mg11 and Mg13 at the nearest Mg position. The final refinement of Mg_44.29_Ru_7_ resulted in an Mg occupancy of 67 (2)% for Mg11 and 33 (2)% for Mg13. Another Mg/Ru phase related to Mg_44.29_Ru_7_ is the Ru-richer phase Mg_43.83_Ru_7.17_ (Westin & Edshammar, 1973 ▸). The main differences between the two phases are: (1) the Ru2 and Ru4 sites are co-occupied by both Ru and Mg in Mg_43.83_Ru_7.17_ while both positions are fully occupied in Mg_44.29_Ru_7_; (2) there is an additional partially occupied Mg1 site in Mg_44.29_Ru_7_ with 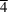
3*m* symmetry; (3) the Mg11 site in Mg_43.83_Ru_7.17_ is split into two separated positions Mg11 and Mg13 in the present model.

**Figure 2:**
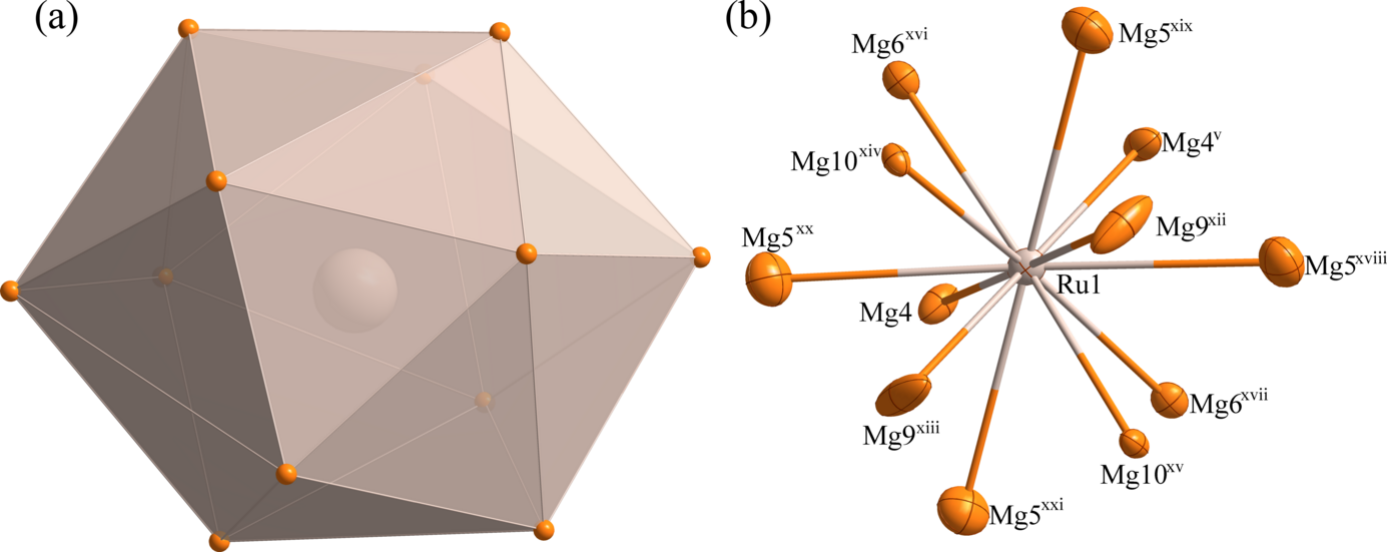
(*a*) The icosa­hedron formed around Ru1 at the 24 *g* site; (*b*) the environment of Ru1 with displacement ellipsoids drawn the 99% probability level. [Symmetry codes: (v) −*x* + 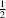, −*y* + 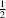, *z*; (xii) −*x* + 1, −*y* + 1, *z*; (xiii) *x* − 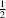, *y* − 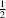, *z*; (xiv) −*x* + 1, *y* − 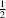, −*z* + 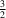; (xv) *x* − 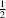, −*y* + 1, −*z* + 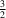; (xvi) *x*, −*y* + 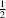, −*z* + 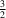; (xvii) −*x* + 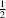, *y*, −*z* + 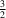; (xviii) −*y* + 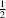, −*z* + 1, *x* + 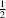; (xix) −*z* + 1, −*x* + 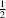, *y* + 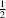; (xx) *y*, *z* − 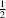, *x* + 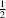; (xxi) *z* − 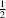, *x*, *y* + 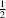.]

The environments of the Ru1, Ru2 and Ru3 and Mg11 sites are shown in Figs. 2 ▸–5 ▸ ▸ ▸, respectively. The Ru1 atom is located at a site with symmetry 2.*mm* (multiplicity 24, Wyckoff letter *g*) and is surrounded by twelve Mg atoms, with the shortest Ru—Mg separation of 2.707 (5) Å for Ru1—Mg4. The Ru2, Ru3 and Mg11 atoms occupy a site with symmetry .3*m* (16 *e*). The Ru2 and Ru3 sites are surrounded by twelve Mg atoms while the Mg11 site is surrounded by thirteen Mg atoms. The shortest Ru—Mg separations are Ru2—Mg3 = 2.778 (4) Å, Ru3—Mg7 = 2.8540 (19) Å and Mg11—Mg5 = 2.993 (11) Å.

## Synthesis and crystallization

High-purity magnesium (indicated purity of 99.9%; 0.1785 g), ruthenium (indicated purity of 99.9%; 0.7421 g) and boron (indicating purity of 99.9%; 0.0793 g) with a stoichiometric ratio of 1:1:1 were evenly mixed and finely ground in agate mortar for 40 min. The mixed powder was then placed in a cemented carbide grinding mold with a diameter of 5 mm and pressed into a block at about 4 MPa for three min. Cylindrical blocks without deformation and cracks were obtained. Details of high-pressure sinter­ing experiments using a six-anvil high-temperature and high-pressure equipment are described elsewhere (Liu & Fan, 2018 ▸). The sample was pressurized to 6 GPa and heated to 1273 K for 40 min, and then quickly cooled to room temperature by turning off the furnace power. A single crystal was selected and mounted on a glass fiber for the X-ray diffraction study.

## Refinement

Crystal data, data collection and structure refinement details are summarized in Table 1 ▸. For better comparison with previously reported Mg_43.83_Ru_7.17_ (Westin & Edshammar, 1973 ▸), the labelling scheme and atomic coordinates of Mg_44.29_Ru_7_ were adapted from it. Site occupancies of Mg1, Mg11 and Mg13 were refined freely. The maximum and minimum residual electron densities in the final difference map of Mg_44.29_Ru_7_ are located 1.80 Å from site Mg2 and 0.98 Å from Ru2, respectively.

## Supplementary Material

Crystal structure: contains datablock(s) I. DOI: 10.1107/S2414314625007291/wm4234sup1.cif

Structure factors: contains datablock(s) I. DOI: 10.1107/S2414314625007291/wm4234Isup2.hkl

modified supplementary materials. DOI: 10.1107/S2414314625007291/wm4234sup4.docx

CCDC reference: 2480571

Additional supporting information:  crystallographic information; 3D view; checkCIF report

## Figures and Tables

**Figure 3:**
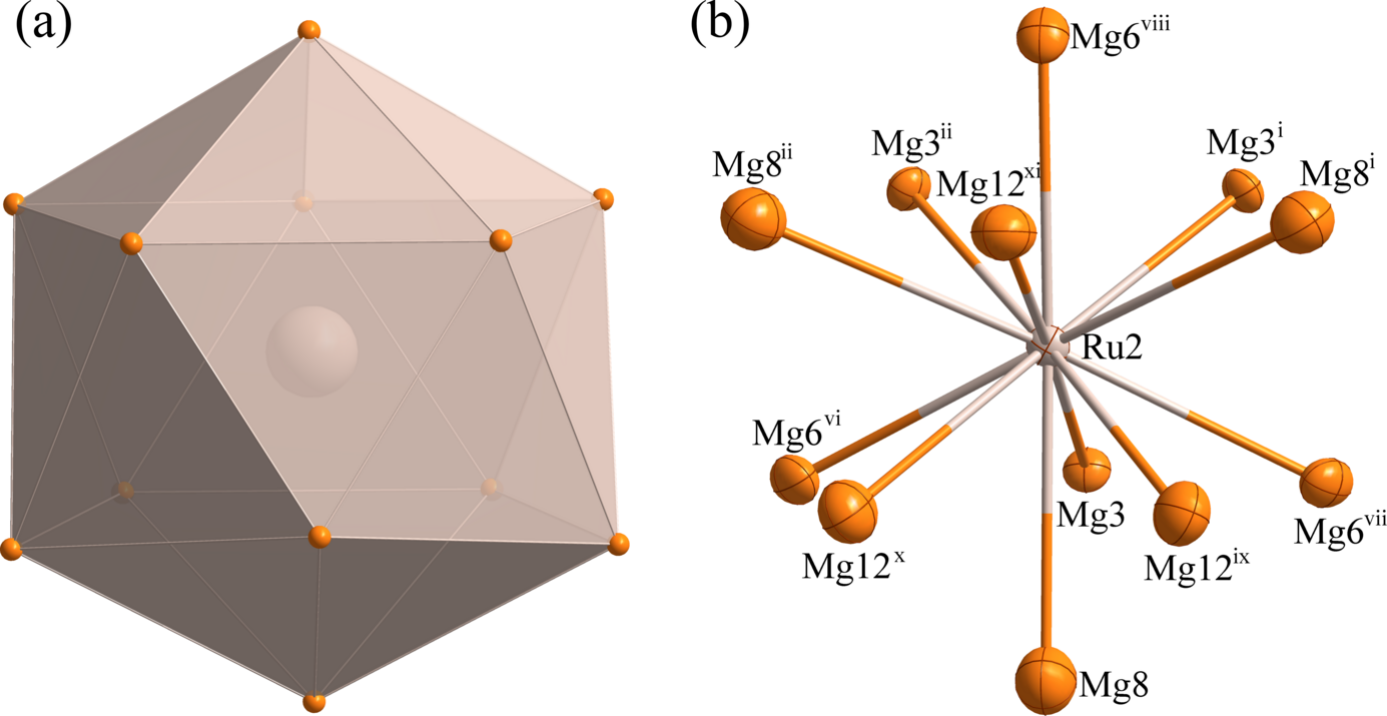
(*a*) The icosa­hedron formed around Ru2 at the 16 *e* site; (*b*) the environment of Ru2 with displacement ellipsoids drawn at the 99% probability level. [Symmetry codes:(i) *y*, *z*, *x*; (ii) *z*, *x*, *y*; (vi) *y*, *z* − 1, *x*; (vii) *z* − 1, *x*, *y*; (viii) *x*, *y*, *z* − 1; (ix) *x* − 1, −*y* + 1, −*z* + 1; (*x*) −*x* + 1, *y* − 1, −*z* + 1; (xi) −*x* + 1, −*y* + 1, *z* − 1.]

**Figure 4:**
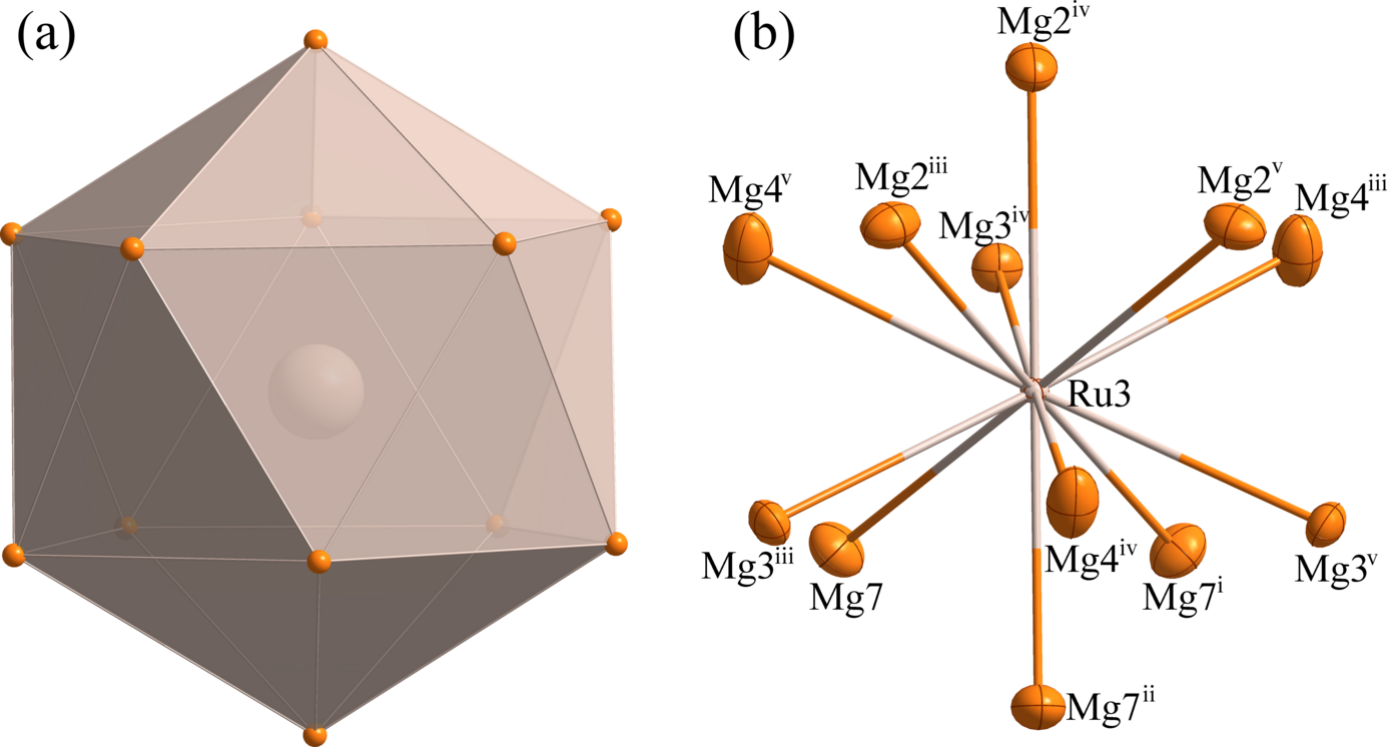
(*a*) The icosa­hedron formed around Ru3 at the 16 *e* site; (*b*) the environment of the Ru3 atom with displacement ellipsoids given at the 99% probability level. [Symmetry codes:(i) *y*, *z*, *x*; (ii) *z*, *x*, *y*; (iii) −*y* + 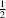, *z*, −*x* + 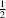; (iv) *z*, −*x* + 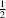, −*y* + 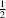; (v) −*x* + 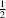, −*y* + 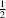, *z*.]

**Figure 5:**
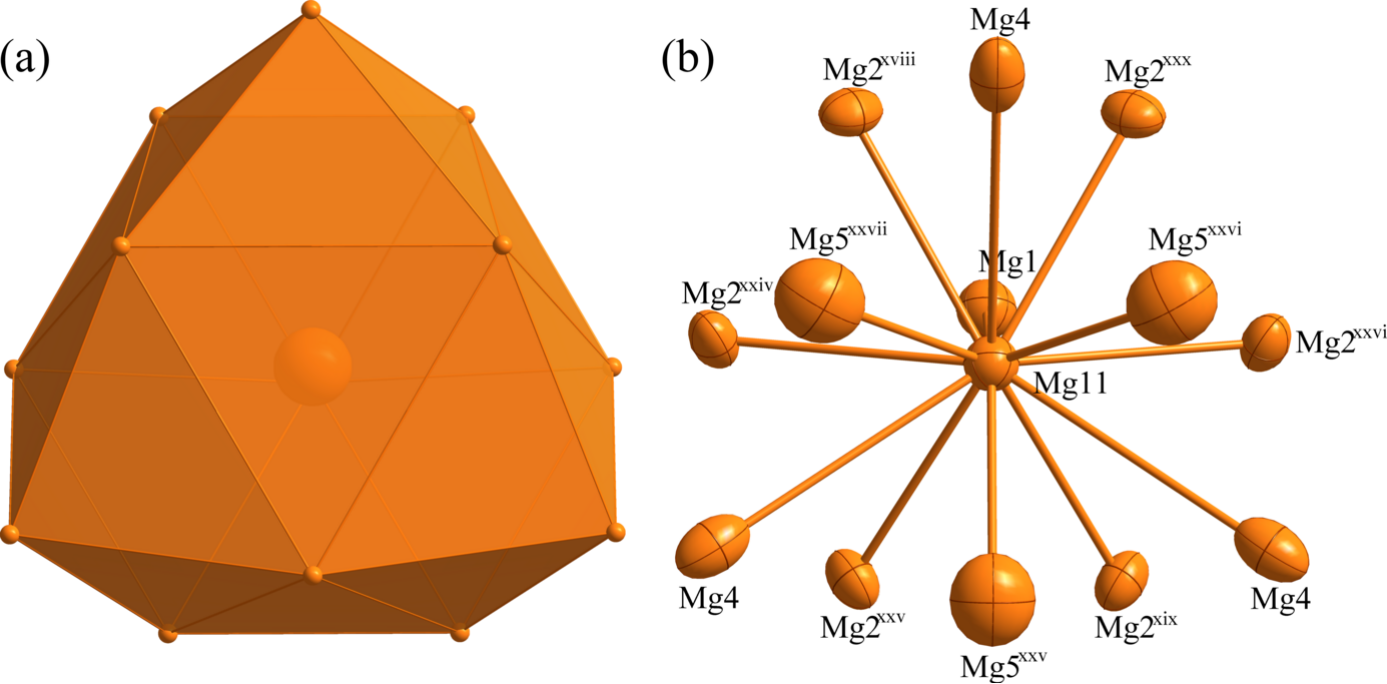
(*a*) The 22-faced polyhedron formed around Mg11 at the 16 *e* site; (*b*) the environment of Mg1 with displacement ellipsoids drawn at the 99% probability level. [Symmetry codes: (xviii) −*y* + 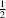, −*z* + 1, *x* + 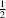; (xix) −*z* + 1, −*x* + 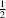, *y* + 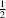; (xxiv) −*z* + 1, *x* + 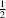, −*y* + 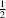; (xxv) *y* + 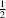, *z*, *x* + 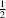; (xxvi) *z*, *x* + 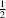, *y* + 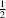; (xxvii) *x* + 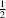, *y* + 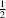, *z*; (xxx) *x* + 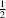, −*y* + 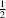, −*z* + 1.]

**Table 1:** Experimental details

Crystal data	
Chemical formula	Mg_44.29_Ru_7_
*M*_r_	1784.30
Crystal system, space group	Cubic, *F* 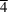 3*m*
Temperature (K)	296
*a* (Å)	20.480 (9)
*V* (Å^3^)	8591 (11)
*Z*	8
Radiation type	Mo *K*α
μ (mm^−1^)	3.05
Crystal size (mm)	0.10 × 0.08 × 0.08

Data collection	
Diffractometer	Bruker D8 Venture Photon 100 CMOS
Absorption correction	Multi-scan (*SADABS*; Krause *et al.*, 2015 ▸)
*T*_min_, *T*_max_	0.623, 0.746
No. of measured, independent and observed [*I* > 2σ(*I*)] reflections	31409, 1021, 903
*R*_int_	0.157
(sin θ/λ)_max_ (Å^−1^)	0.645

Refinement	
*R*[*F*^2^ > 2σ(*F*^2^)], *wR*(*F*^2^), *S*	0.040, 0.063, 1.06
No. of reflections	1021
No. of parameters	65
Δρ_max_, Δρ_min_ (e Å^−3^)	0.72, −0.81
Absolute structure	Flack *x* determined using 359 quotients [(*I*^+^)−(*I*^−^)]/[(*I*^+^)+(*I*^−^)] (Parsons *et al.*, 2013 ▸)
Absolute structure parameter	−0.05 (5)

Computer programs: *APEX3* and *SAINT* (Bruker, 2023 ▸), *SHELXT* (Sheldrick, 2015*a* ▸), *SHELXL* (Sheldrick, 2015*b* ▸), *DIAMOND* (Brandenburg & Putz, 2017 ▸) and *publCIF* (Westrip, 2010 ▸).

## full crystallographic data

### Tetratetracontamagnesium heptaruthenium Crystal data

**Table:**

Mg_44.29_Ru_7_	Mo *K*α radiation, λ = 0.71073 Å
*M**_r_* = 1784.30	Cell parameters from 5950 reflections
Cubic, *F*43*m*	θ = 2.8–26.2°
*a* = 20.480 (9) Å	µ = 3.05 mm^−^^1^
*V* = 8591 (11) Å^3^	*T* = 296 K
*Z* = 8	Lump, gray
*F*(000) = 6716	0.10 × 0.08 × 0.08 mm
*D*_x_ = 2.759 Mg m^−^^3^	

### Tetratetracontamagnesium heptaruthenium Data collection

**Table:**

Bruker D8 Venture Photon 100 CMOS diffractometer	903 reflections with *I* > 2σ(*I*)
phi and ω scans	*R*_int_ = 0.157
Absorption correction: multi-scan (*SADABS*; Krause *et al.*, 2015)	θ_max_ = 27.3°, θ_min_ = 2.8°
*T*_min_ = 0.623, *T*_max_ = 0.746	*h* = −26→26
31409 measured reflections	*k* = −26→21
1021 independent reflections	*l* = −26→26

### Tetratetracontamagnesium heptaruthenium Refinement

**Table:**

Refinement on *F*^2^	0 restraints
Least-squares matrix: full	*w* = 1/[σ^2^(*F*_o_^2^) + (0.0178*P*)^2^ + 174.1245*P*] where *P* = (*F*_o_^2^ + 2*F*_c_^2^)/3
*R*[*F*^2^ > 2σ(*F*^2^)] = 0.040	(Δ/σ)_max_ < 0.001
*wR*(*F*^2^) = 0.063	Δρ_max_ = 0.72 e Å^−^^3^
*S* = 1.06	Δρ_min_ = −0.81 e Å^−^^3^
1021 reflections	Absolute structure: Flack *x* determined using 359 quotients [(*I*^+^)-(*I*^-^)]/[(*I*^+^)+(*I*^-^)] (Parsons *et al.*, 2013)
65 parameters	Absolute structure parameter: −0.05 (5)

### Tetratetracontamagnesium heptaruthenium Special details

**Table:**

Geometry. All esds (except the esd in the dihedral angle between two l.s. planes) are estimated using the full covariance matrix. The cell esds are taken into account individually in the estimation of esds in distances, angles and torsion angles; correlations between esds in cell parameters are only used when they are defined by crystal symmetry. An approximate (isotropic) treatment of cell esds is used for estimating esds involving l.s. planes.

### Tetratetracontamagnesium heptaruthenium Fractional atomic coordinates and isotropic or equivalent isotropic displacement parameters (Å^2^)

**Table:**

	*x*	*y*	*z*	*U*_iso_*/*U*_eq_	Occ. (<1)
Ru1	0.250000	0.250000	0.58281 (7)	0.0136 (4)	
Ru2	0.08650 (5)	0.08650 (5)	0.08650 (5)	0.0079 (4)	
Ru3	0.34837 (5)	0.34837 (5)	0.34837 (5)	0.0032 (3)	
Mg1	0.500000	0.500000	0.500000	0.014 (10)	0.59 (6)
Mg2	0.05113 (14)	0.05113 (14)	0.3415 (2)	0.0126 (10)	
Mg3	0.10802 (14)	0.10802 (14)	0.2187 (2)	0.0092 (9)	
Mg4	0.19177 (16)	0.19177 (16)	0.4794 (2)	0.0164 (10)	
Mg5	0.09229 (18)	0.09229 (18)	0.7261 (2)	0.0266 (13)	
Mg6	0.15752 (17)	0.15752 (17)	0.9815 (2)	0.0133 (11)	
Mg7	0.250000	0.250000	0.3563 (3)	0.0127 (12)	
Mg8	0.000000	0.000000	0.1817 (4)	0.0207 (17)	
Mg9	0.6878 (4)	0.6878 (4)	0.6878 (4)	0.034 (3)	
Mg10	0.8320 (2)	0.8320 (2)	0.8320 (2)	0.0103 (18)	
Mg11	0.5809 (4)	0.5809 (4)	0.5809 (4)	0.012 (2)	0.671 (19)
Mg12	0.9447 (2)	0.9447 (2)	0.9447 (2)	0.019 (2)	
Mg13	0.5583 (9)	0.5583 (9)	0.5583 (9)	0.012 (2)	0.329 (19)

### Tetratetracontamagnesium heptaruthenium Atomic displacement parameters (Å^2^)

**Table:**

	*U*^11^	*U*^22^	*U*^33^	*U*^12^	*U*^13^	*U*^23^
Mg1	0.014 (10)	0.014 (10)	0.014 (10)	0.000	0.000	0.000
Ru1	0.0160 (5)	0.0160 (5)	0.0088 (8)	0.0014 (8)	0.000	0.000
Ru2	0.0079 (4)	0.0079 (4)	0.0079 (4)	−0.0011 (5)	−0.0011 (5)	−0.0011 (5)
Ru3	0.0032 (3)	0.0032 (3)	0.0032 (3)	−0.0002 (4)	−0.0002 (4)	−0.0002 (4)
Mg2	0.0112 (14)	0.0112 (14)	0.015 (2)	−0.0001 (17)	0.0029 (13)	0.0029 (13)
Mg3	0.0106 (13)	0.0106 (13)	0.006 (2)	−0.0004 (16)	−0.0009 (12)	−0.0009 (12)
Mg4	0.0166 (15)	0.0166 (15)	0.016 (3)	0.005 (2)	−0.0056 (15)	−0.0056 (15)
Mg5	0.0249 (18)	0.0249 (18)	0.030 (3)	−0.003 (2)	−0.0044 (16)	−0.0044 (16)
Mg6	0.0153 (16)	0.0153 (16)	0.009 (2)	0.002 (2)	−0.0010 (13)	−0.0010 (13)
Mg7	0.0106 (17)	0.0106 (17)	0.017 (3)	−0.002 (3)	0.000	0.000
Mg8	0.021 (2)	0.021 (2)	0.021 (4)	0.003 (3)	0.000	0.000
Mg9	0.034 (3)	0.034 (3)	0.034 (3)	0.021 (3)	0.021 (3)	0.021 (3)
Mg10	0.0103 (18)	0.0103 (18)	0.0103 (18)	0.0023 (15)	0.0023 (15)	0.0023 (15)
Mg11	0.012 (2)	0.012 (2)	0.012 (2)	0.000 (3)	0.000 (3)	0.000 (3)
Mg12	0.019 (2)	0.019 (2)	0.019 (2)	−0.002 (2)	−0.002 (2)	−0.002 (2)
Mg13	0.012 (2)	0.012 (2)	0.012 (2)	0.000 (3)	0.000 (3)	0.000 (3)

### Tetratetracontamagnesium heptaruthenium Geometric parameters (Å, º)

**Table:**

Ru1—Mg4	2.707 (5)	Mg4—Mg2^xxvi^	3.025 (5)
Ru1—Mg4^i^	2.707 (5)	Mg4—Mg2^xxvii^	3.025 (5)
Ru1—Mg9^ii^	2.8050 (19)	Mg4—Mg5^x^	3.160 (4)
Ru1—Mg9^iii^	2.8050 (19)	Mg4—Mg5^xi^	3.160 (4)
Ru1—Mg10^iv^	2.947 (3)	Mg4—Mg4^i^	3.373 (10)
Ru1—Mg10^v^	2.947 (3)	Mg13—Mg2^xxix^	3.042 (4)
Ru1—Mg6^vi^	2.985 (5)	Mg13—Mg2^xxiv^	3.042 (4)
Ru1—Mg6^vii^	2.985 (5)	Mg13—Mg2^ix^	3.042 (4)
Ru1—Mg5^viii^	3.273 (4)	Mg13—Mg2^xxx^	3.042 (4)
Ru1—Mg5^ix^	3.273 (4)	Mg13—Mg2^xxxi^	3.042 (4)
Ru1—Mg5^x^	3.273 (4)	Mg13—Mg13^ii^	3.38 (5)
Ru1—Mg5^xi^	3.273 (4)	Mg13—Mg13^xxii^	3.38 (5)
Ru2—Mg3^xii^	2.778 (4)	Mg13—Mg13^xxiii^	3.38 (5)
Ru2—Mg3^xiii^	2.778 (4)	Mg13—Mg5^xxxii^	3.58 (2)
Ru2—Mg3	2.778 (4)	Mg13—Mg5^xxxiii^	3.58 (2)
Ru2—Mg6^xiv^	2.976 (5)	Mg5—Mg9^iii^	2.876 (9)
Ru2—Mg6^xv^	2.976 (5)	Mg6—Mg12^ii^	3.054 (7)
Ru2—Mg6^xvi^	2.976 (5)	Mg6—Mg10^ii^	3.075 (7)
Ru2—Mg12^xvii^	3.042 (3)	Mg6—Mg5^xxxiv^	3.122 (5)
Ru2—Mg12^xviii^	3.042 (3)	Mg6—Mg5^xxxv^	3.122 (5)
Ru2—Mg12^xix^	3.042 (3)	Mg6—Mg4^vi^	3.265 (5)
Ru2—Mg8	3.175 (5)	Mg6—Mg4^vii^	3.265 (5)
Ru2—Mg8^xii^	3.175 (5)	Mg6—Mg8^xxxvi^	3.286 (4)
Ru2—Mg8^xiii^	3.175 (5)	Mg6—Mg8^xxxvii^	3.286 (4)
Ru3—Mg7^xii^	2.8540 (19)	Mg7—Mg4^i^	3.033 (6)
Ru3—Mg7^xiii^	2.8540 (19)	Mg7—Mg4	3.033 (6)
Ru3—Mg7	2.8540 (19)	Mg7—Mg3^xx^	3.066 (4)
Ru3—Mg2^xx^	2.914 (4)	Mg7—Mg3^xxi^	3.066 (4)
Ru3—Mg2^xxi^	2.914 (4)	Mg7—Mg3^xxvi^	3.066 (4)
Ru3—Mg2^i^	2.914 (4)	Mg7—Mg3^xxvii^	3.066 (4)
Ru3—Mg4^xx^	2.925 (5)	Mg7—Mg7^xxvi^	3.080 (8)
Ru3—Mg4^i^	2.925 (5)	Mg7—Mg7^xxxviii^	3.080 (8)
Ru3—Mg4^xxi^	2.925 (5)	Mg7—Mg7^xiii^	3.080 (8)
Ru3—Mg3^i^	2.941 (4)	Mg7—Mg7^xii^	3.080 (8)
Ru3—Mg3^xx^	2.941 (4)	Mg8—Mg12^xvii^	3.045 (7)
Ru3—Mg3^xxi^	2.941 (4)	Mg8—Mg12^xviii^	3.045 (7)
Mg1—Mg13^xxii^	2.07 (3)	Mg8—Mg5^xxviii^	3.272 (8)
Mg1—Mg13^xxiii^	2.07 (3)	Mg8—Mg5^xxxix^	3.272 (8)
Mg1—Mg13^ii^	2.07 (3)	Mg9—Mg9^xl^	3.60 (2)
Mg1—Mg13	2.07 (3)	Mg9—Mg9^xli^	3.60 (2)
Mg1—Mg11^xxiii^	2.869 (15)	Mg9—Mg9^xlii^	3.60 (2)
Mg1—Mg11^xxii^	2.869 (15)	Mg10—Mg9^xlii^	3.009 (9)
Mg1—Mg11^ii^	2.869 (15)	Mg10—Mg9^xli^	3.009 (9)
Mg1—Mg11	2.869 (15)	Mg10—Mg9^xl^	3.009 (9)
Mg1—Mg2^xxi^	3.568 (5)	Mg10—Mg5^xliii^	3.084 (5)
Mg1—Mg2^i^	3.568 (4)	Mg10—Mg5^xliv^	3.084 (5)
Mg1—Mg2^xx^	3.568 (5)	Mg10—Mg5^ii^	3.084 (5)
Mg1—Mg2^xxiv^	3.568 (5)	Mg11—Mg5^xxxii^	2.993 (11)
Mg2—Mg2^xxv^	2.962 (8)	Mg11—Mg5^xxxiii^	2.993 (11)
Mg2—Mg2^xxvi^	3.110 (8)	Mg11—Mg5^xlv^	2.993 (11)
Mg2—Mg2^xxvii^	3.110 (8)	Mg11—Mg2^viii^	3.195 (5)
Mg2—Mg5^xxviii^	3.355 (5)	Mg11—Mg2^xxx^	3.195 (5)
Mg3—Mg2	3.007 (6)	Mg11—Mg2^xxix^	3.195 (5)
Mg3—Mg6^xiv^	3.052 (4)	Mg11—Mg2^xxiv^	3.195 (5)
Mg3—Mg6^xv^	3.052 (4)	Mg11—Mg2^ix^	3.195 (5)
Mg3—Mg4^xxvi^	3.085 (3)	Mg11—Mg2^xxxi^	3.195 (5)
Mg3—Mg4^xxvii^	3.085 (3)	Mg12—Mg12^xlvi^	3.205 (14)
Mg3—Mg3^xii^	3.205 (7)	Mg12—Mg12^xlvii^	3.205 (14)
Mg3—Mg3^xiii^	3.205 (7)	Mg12—Mg12^xlviii^	3.205 (14)
Mg3—Mg8	3.219 (5)	Mg13—Mg2^viii^	3.042 (4)
			
Mg7^xii^—Ru3—Mg7^xiii^	65.3 (2)	Ru2^xlix^—Mg6—Mg5^xxxv^	120.91 (13)
Mg7^xii^—Ru3—Mg7	65.3 (2)	Ru1^vii^—Mg6—Mg5^xxxv^	64.76 (13)
Mg7^xiii^—Ru3—Mg7	65.3 (2)	Mg3^xxxvii^—Mg6—Mg5^xxxv^	87.45 (12)
Mg7^xii^—Ru3—Mg2^xx^	179.49 (17)	Mg3^xxxvi^—Mg6—Mg5^xxxv^	147.16 (15)
Mg7^xiii^—Ru3—Mg2^xx^	115.10 (11)	Mg12^ii^—Mg6—Mg5^xxxv^	96.34 (13)
Mg7—Ru3—Mg2^xx^	115.09 (11)	Mg10^ii^—Mg6—Mg5^xxxv^	59.68 (12)
Mg7^xii^—Ru3—Mg2^xxi^	115.10 (11)	Mg5^xxxiv^—Mg6—Mg5^xxxv^	114.8 (2)
Mg7^xiii^—Ru3—Mg2^xxi^	179.49 (17)	Ru2^xlix^—Mg6—Mg4^vi^	112.95 (13)
Mg7—Ru3—Mg2^xxi^	115.09 (11)	Ru1^vii^—Mg6—Mg4^vi^	51.09 (10)
Mg2^xx^—Ru3—Mg2^xxi^	64.50 (14)	Mg3^xxxvii^—Mg6—Mg4^vi^	58.35 (10)
Mg7^xii^—Ru3—Mg2^i^	115.10 (11)	Mg3^xxxvi^—Mg6—Mg4^vi^	91.02 (15)
Mg7^xiii^—Ru3—Mg2^i^	115.10 (11)	Mg12^ii^—Mg6—Mg4^vi^	148.82 (9)
Mg7—Ru3—Mg2^i^	179.49 (17)	Mg10^ii^—Mg6—Mg4^vi^	99.40 (18)
Mg2^xx^—Ru3—Mg2^i^	64.50 (14)	Mg5^xxxiv^—Mg6—Mg4^vi^	111.05 (18)
Mg2^xxi^—Ru3—Mg2^i^	64.50 (14)	Mg5^xxxv^—Mg6—Mg4^vi^	59.26 (11)
Mg7^xii^—Ru3—Mg4^xx^	63.31 (14)	Ru2^xlix^—Mg6—Mg4^vii^	112.95 (13)
Mg7^xiii^—Ru3—Mg4^xx^	117.74 (9)	Ru1^vii^—Mg6—Mg4^vii^	51.09 (10)
Mg7—Ru3—Mg4^xx^	117.74 (9)	Mg3^xxxvii^—Mg6—Mg4^vii^	91.02 (15)
Mg2^xx^—Ru3—Mg4^xx^	116.19 (14)	Mg3^xxxvi^—Mg6—Mg4^vii^	58.35 (10)
Mg2^xxi^—Ru3—Mg4^xx^	62.41 (8)	Mg12^ii^—Mg6—Mg4^vii^	148.82 (9)
Mg2^i^—Ru3—Mg4^xx^	62.41 (8)	Mg10^ii^—Mg6—Mg4^vii^	99.40 (18)
Mg7^xii^—Ru3—Mg4^i^	117.74 (9)	Mg5^xxxiv^—Mg6—Mg4^vii^	59.26 (11)
Mg7^xiii^—Ru3—Mg4^i^	117.74 (9)	Mg5^xxxv^—Mg6—Mg4^vii^	111.05 (18)
Mg7—Ru3—Mg4^i^	63.31 (14)	Mg4^vi^—Mg6—Mg4^vii^	62.21 (17)
Mg2^xx^—Ru3—Mg4^i^	62.41 (8)	Ru2^xlix^—Mg6—Mg8^xxxvi^	60.69 (13)
Mg2^xxi^—Ru3—Mg4^i^	62.41 (8)	Ru1^vii^—Mg6—Mg8^xxxvi^	125.32 (15)
Mg2^i^—Ru3—Mg4^i^	116.19 (14)	Mg3^xxxvii^—Mg6—Mg8^xxxvi^	110.80 (15)
Mg4^xx^—Ru3—Mg4^i^	115.90 (7)	Mg3^xxxvi^—Mg6—Mg8^xxxvi^	60.91 (10)
Mg7^xii^—Ru3—Mg4^xxi^	117.74 (9)	Mg12^ii^—Mg6—Mg8^xxxvi^	57.26 (15)
Mg7^xiii^—Ru3—Mg4^xxi^	63.31 (14)	Mg10^ii^—Mg6—Mg8^xxxvi^	99.94 (11)
Mg7—Ru3—Mg4^xxi^	117.74 (9)	Mg5^xxxiv^—Mg6—Mg8^xxxvi^	61.35 (16)
Mg2^xx^—Ru3—Mg4^xxi^	62.41 (8)	Mg5^xxxv^—Mg6—Mg8^xxxvi^	150.45 (17)
Mg2^xxi^—Ru3—Mg4^xxi^	116.19 (14)	Mg4^vi^—Mg6—Mg8^xxxvi^	150.16 (17)
Mg2^i^—Ru3—Mg4^xxi^	62.41 (8)	Mg4^vii^—Mg6—Mg8^xxxvi^	92.29 (17)
Mg4^xx^—Ru3—Mg4^xxi^	115.90 (7)	Ru2^xlix^—Mg6—Mg8^xxxvii^	60.69 (13)
Mg4^i^—Ru3—Mg4^xxi^	115.90 (7)	Ru1^vii^—Mg6—Mg8^xxxvii^	125.32 (15)
Mg7^xii^—Ru3—Mg3^i^	63.86 (7)	Mg3^xxxvii^—Mg6—Mg8^xxxvii^	60.91 (10)
Mg7^xiii^—Ru3—Mg3^i^	63.86 (7)	Mg3^xxxvi^—Mg6—Mg8^xxxvii^	110.80 (15)
Mg7—Ru3—Mg3^i^	118.71 (17)	Mg12^ii^—Mg6—Mg8^xxxvii^	57.26 (15)
Mg2^xx^—Ru3—Mg3^i^	115.98 (8)	Mg10^ii^—Mg6—Mg8^xxxvii^	99.94 (11)
Mg2^xxi^—Ru3—Mg3^i^	115.98 (8)	Mg5^xxxiv^—Mg6—Mg8^xxxvii^	150.45 (17)
Mg2^i^—Ru3—Mg3^i^	61.80 (12)	Mg5^xxxv^—Mg6—Mg8^xxxvii^	61.35 (16)
Mg4^xx^—Ru3—Mg3^i^	63.44 (4)	Mg4^vi^—Mg6—Mg8^xxxvii^	92.29 (17)
Mg4^i^—Ru3—Mg3^i^	177.99 (15)	Mg4^vii^—Mg6—Mg8^xxxvii^	150.16 (17)
Mg4^xxi^—Ru3—Mg3^i^	63.44 (4)	Mg8^xxxvi^—Mg6—Mg8^xxxvii^	106.4 (3)
Mg7^xii^—Ru3—Mg3^xx^	118.71 (17)	Ru1^l^—Mg10—Ru1^li^	110.46 (15)
Mg7^xiii^—Ru3—Mg3^xx^	63.86 (7)	Ru1^l^—Mg10—Ru1^lii^	110.46 (15)
Mg7—Ru3—Mg3^xx^	63.86 (7)	Ru1^li^—Mg10—Ru1^lii^	110.46 (15)
Mg2^xx^—Ru3—Mg3^xx^	61.80 (12)	Ru1^l^—Mg10—Mg9^xlii^	115.2 (3)
Mg2^xxi^—Ru3—Mg3^xx^	115.98 (8)	Ru1^li^—Mg10—Mg9^xlii^	56.17 (10)
Mg2^i^—Ru3—Mg3^xx^	115.98 (8)	Ru1^lii^—Mg10—Mg9^xlii^	56.17 (10)
Mg4^xx^—Ru3—Mg3^xx^	177.99 (15)	Ru1^l^—Mg10—Mg9^xli^	56.17 (10)
Mg4^i^—Ru3—Mg3^xx^	63.44 (4)	Ru1^li^—Mg10—Mg9^xli^	115.2 (3)
Mg4^xxi^—Ru3—Mg3^xx^	63.44 (4)	Ru1^lii^—Mg10—Mg9^xli^	56.17 (10)
Mg3^i^—Ru3—Mg3^xx^	117.15 (5)	Mg9^xlii^—Mg10—Mg9^xli^	73.5 (4)
Mg7^xii^—Ru3—Mg3^xxi^	63.86 (7)	Ru1^l^—Mg10—Mg9^xl^	56.17 (10)
Mg7^xiii^—Ru3—Mg3^xxi^	118.71 (17)	Ru1^li^—Mg10—Mg9^xl^	56.17 (10)
Mg7—Ru3—Mg3^xxi^	63.86 (7)	Ru1^lii^—Mg10—Mg9^xl^	115.2 (3)
Mg2^xx^—Ru3—Mg3^xxi^	115.98 (8)	Mg9^xlii^—Mg10—Mg9^xl^	73.5 (4)
Mg2^xxi^—Ru3—Mg3^xxi^	61.80 (12)	Mg9^xli^—Mg10—Mg9^xl^	73.5 (4)
Mg2^i^—Ru3—Mg3^xxi^	115.98 (8)	Ru1^l^—Mg10—Mg6^xliii^	59.37 (10)
Mg4^xx^—Ru3—Mg3^xxi^	63.44 (4)	Ru1^li^—Mg10—Mg6^xliii^	124.46 (11)
Mg4^i^—Ru3—Mg3^xxi^	63.44 (4)	Ru1^lii^—Mg10—Mg6^xliii^	124.46 (11)
Mg4^xxi^—Ru3—Mg3^xxi^	177.99 (15)	Mg9^xlii^—Mg10—Mg6^xliii^	174.6 (3)
Mg3^i^—Ru3—Mg3^xxi^	117.15 (5)	Mg9^xli^—Mg10—Mg6^xliii^	102.25 (17)
Mg3^xx^—Ru3—Mg3^xxi^	117.15 (5)	Mg9^xl^—Mg10—Mg6^xliii^	102.25 (17)
Mg3^xii^—Ru2—Mg3^xiii^	70.46 (14)	Ru1^l^—Mg10—Mg6^xliv^	124.46 (11)
Mg3^xii^—Ru2—Mg3	70.46 (14)	Ru1^li^—Mg10—Mg6^xliv^	124.46 (11)
Mg3^xiii^—Ru2—Mg3	70.46 (14)	Ru1^lii^—Mg10—Mg6^xliv^	59.37 (10)
Mg3^xii^—Ru2—Mg6^xiv^	123.31 (13)	Mg9^xlii^—Mg10—Mg6^xliv^	102.25 (17)
Mg3^xiii^—Ru2—Mg6^xiv^	63.95 (7)	Mg9^xli^—Mg10—Mg6^xliv^	102.25 (17)
Mg3—Ru2—Mg6^xiv^	63.95 (7)	Mg9^xl^—Mg10—Mg6^xliv^	174.6 (3)
Mg3^xii^—Ru2—Mg6^xv^	63.95 (7)	Mg6^xliii^—Mg10—Mg6^xliv^	81.8 (2)
Mg3^xiii^—Ru2—Mg6^xv^	123.31 (13)	Ru1^l^—Mg10—Mg6^ii^	124.46 (11)
Mg3—Ru2—Mg6^xv^	63.95 (7)	Ru1^li^—Mg10—Mg6^ii^	59.37 (10)
Mg6^xiv^—Ru2—Mg6^xv^	117.88 (4)	Ru1^lii^—Mg10—Mg6^ii^	124.46 (11)
Mg3^xii^—Ru2—Mg6^xvi^	63.95 (7)	Mg9^xlii^—Mg10—Mg6^ii^	102.25 (17)
Mg3^xiii^—Ru2—Mg6^xvi^	63.95 (7)	Mg9^xli^—Mg10—Mg6^ii^	174.6 (3)
Mg3—Ru2—Mg6^xvi^	123.31 (13)	Mg9^xl^—Mg10—Mg6^ii^	102.25 (17)
Mg6^xiv^—Ru2—Mg6^xvi^	117.88 (4)	Mg6^xliii^—Mg10—Mg6^ii^	81.8 (2)
Mg6^xv^—Ru2—Mg6^xvi^	117.88 (4)	Mg6^xliv^—Mg10—Mg6^ii^	81.8 (2)
Mg3^xii^—Ru2—Mg12^xvii^	112.91 (14)	Ru1^l^—Mg10—Mg5^xliii^	171.6 (3)
Mg3^xiii^—Ru2—Mg12^xvii^	175.70 (18)	Ru1^li^—Mg10—Mg5^xliii^	65.68 (5)
Mg3—Ru2—Mg12^xvii^	112.91 (14)	Ru1^lii^—Mg10—Mg5^xliii^	65.68 (5)
Mg6^xiv^—Ru2—Mg12^xvii^	114.68 (9)	Mg9^xlii^—Mg10—Mg5^xliii^	56.3 (2)
Mg6^xv^—Ru2—Mg12^xvii^	60.99 (17)	Mg9^xli^—Mg10—Mg5^xliii^	117.78 (16)
Mg6^xvi^—Ru2—Mg12^xvii^	114.68 (9)	Mg9^xl^—Mg10—Mg5^xliii^	117.78 (16)
Mg3^xii^—Ru2—Mg12^xviii^	175.70 (18)	Mg6^xliii^—Mg10—Mg5^xliii^	129.1 (3)
Mg3^xiii^—Ru2—Mg12^xviii^	112.91 (14)	Mg6^xliv^—Mg10—Mg5^xliii^	60.92 (10)
Mg3—Ru2—Mg12^xviii^	112.91 (14)	Mg6^ii^—Mg10—Mg5^xliii^	60.92 (10)
Mg6^xiv^—Ru2—Mg12^xviii^	60.99 (17)	Ru1^l^—Mg10—Mg5^xliv^	65.68 (5)
Mg6^xv^—Ru2—Mg12^xviii^	114.68 (9)	Ru1^li^—Mg10—Mg5^xliv^	65.68 (5)
Mg6^xvi^—Ru2—Mg12^xviii^	114.68 (9)	Ru1^lii^—Mg10—Mg5^xliv^	171.6 (3)
Mg12^xvii^—Ru2—Mg12^xviii^	63.6 (2)	Mg9^xlii^—Mg10—Mg5^xliv^	117.78 (16)
Mg3^xii^—Ru2—Mg12^xix^	112.91 (14)	Mg9^xli^—Mg10—Mg5^xliv^	117.78 (16)
Mg3^xiii^—Ru2—Mg12^xix^	112.91 (14)	Mg9^xl^—Mg10—Mg5^xliv^	56.3 (2)
Mg3—Ru2—Mg12^xix^	175.70 (18)	Mg6^xliii^—Mg10—Mg5^xliv^	60.92 (10)
Mg6^xiv^—Ru2—Mg12^xix^	114.68 (9)	Mg6^xliv^—Mg10—Mg5^xliv^	129.1 (3)
Mg6^xv^—Ru2—Mg12^xix^	114.68 (9)	Mg6^ii^—Mg10—Mg5^xliv^	60.92 (10)
Mg6^xvi^—Ru2—Mg12^xix^	60.99 (17)	Mg5^xliii^—Mg10—Mg5^xliv^	117.04 (10)
Mg12^xvii^—Ru2—Mg12^xix^	63.6 (2)	Ru1^l^—Mg10—Mg5^ii^	65.68 (5)
Mg12^xviii^—Ru2—Mg12^xix^	63.6 (2)	Ru1^li^—Mg10—Mg5^ii^	171.6 (3)
Mg3^xii^—Ru2—Mg8	122.33 (9)	Ru1^lii^—Mg10—Mg5^ii^	65.68 (5)
Mg3^xiii^—Ru2—Mg8	122.33 (9)	Mg9^xlii^—Mg10—Mg5^ii^	117.78 (16)
Mg3—Ru2—Mg8	65.07 (14)	Mg9^xli^—Mg10—Mg5^ii^	56.3 (2)
Mg6^xiv^—Ru2—Mg8	64.48 (5)	Mg9^xl^—Mg10—Mg5^ii^	117.78 (16)
Mg6^xv^—Ru2—Mg8	64.48 (5)	Mg6^xliii^—Mg10—Mg5^ii^	60.92 (10)
Mg6^xvi^—Ru2—Mg8	171.62 (15)	Mg6^xliv^—Mg10—Mg5^ii^	60.92 (10)
Mg12^xvii^—Ru2—Mg8	58.60 (10)	Mg6^ii^—Mg10—Mg5^ii^	129.1 (3)
Mg12^xviii^—Ru2—Mg8	58.60 (10)	Mg5^xliii^—Mg10—Mg5^ii^	117.04 (10)
Mg12^xix^—Ru2—Mg8	110.63 (19)	Mg5^xliv^—Mg10—Mg5^ii^	117.04 (10)
Mg3^xii^—Ru2—Mg8^xii^	65.06 (14)	Ru1—Mg4—Ru3^i^	164.87 (19)
Mg3^xiii^—Ru2—Mg8^xii^	122.33 (9)	Ru1—Mg4—Mg2^xxvi^	132.89 (14)
Mg3—Ru2—Mg8^xii^	122.33 (9)	Ru3^i^—Mg4—Mg2^xxvi^	58.61 (11)
Mg6^xiv^—Ru2—Mg8^xii^	171.62 (15)	Ru1—Mg4—Mg2^xxvii^	132.89 (14)
Mg6^xv^—Ru2—Mg8^xii^	64.48 (5)	Ru3^i^—Mg4—Mg2^xxvii^	58.61 (11)
Mg6^xvi^—Ru2—Mg8^xii^	64.48 (5)	Mg2^xxvi^—Mg4—Mg2^xxvii^	61.86 (19)
Mg12^xvii^—Ru2—Mg8^xii^	58.60 (10)	Ru1—Mg4—Mg7	107.67 (16)
Mg12^xviii^—Ru2—Mg8^xii^	110.63 (19)	Ru3^i^—Mg4—Mg7	57.20 (10)
Mg12^xix^—Ru2—Mg8^xii^	58.60 (10)	Mg2^xxvi^—Mg4—Mg7	106.91 (15)
Mg8—Ru2—Mg8^xii^	111.97 (10)	Mg2^xxvii^—Mg4—Mg7	106.91 (15)
Mg3^xii^—Ru2—Mg8^xiii^	122.33 (9)	Ru1—Mg4—Mg3^xxvi^	115.92 (13)
Mg3^xiii^—Ru2—Mg8^xiii^	65.06 (14)	Ru3^i^—Mg4—Mg3^xxvi^	58.53 (11)
Mg3—Ru2—Mg8^xiii^	122.33 (9)	Mg2^xxvi^—Mg4—Mg3^xxvi^	58.96 (11)
Mg6^xiv^—Ru2—Mg8^xiii^	64.48 (5)	Mg2^xxvii^—Mg4—Mg3^xxvi^	108.70 (18)
Mg6^xv^—Ru2—Mg8^xiii^	171.62 (15)	Mg7—Mg4—Mg3^xxvi^	60.14 (11)
Mg6^xvi^—Ru2—Mg8^xiii^	64.48 (5)	Ru1—Mg4—Mg3^xxvii^	115.92 (13)
Mg12^xvii^—Ru2—Mg8^xiii^	110.63 (19)	Ru3^i^—Mg4—Mg3^xxvii^	58.53 (11)
Mg12^xviii^—Ru2—Mg8^xiii^	58.60 (10)	Mg2^xxvi^—Mg4—Mg3^xxvii^	108.70 (18)
Mg12^xix^—Ru2—Mg8^xiii^	58.60 (10)	Mg2^xxvii^—Mg4—Mg3^xxvii^	58.96 (11)
Mg8—Ru2—Mg8^xiii^	111.97 (10)	Mg7—Mg4—Mg3^xxvii^	60.14 (11)
Mg8^xii^—Ru2—Mg8^xiii^	111.97 (10)	Mg3^xxvi^—Mg4—Mg3^xxvii^	108.9 (2)
Mg4—Ru1—Mg4^i^	77.09 (19)	Ru1—Mg4—Mg5^x^	67.29 (13)
Mg4—Ru1—Mg9^ii^	178.6 (3)	Ru3^i^—Mg4—Mg5^x^	123.54 (15)
Mg4^i^—Ru1—Mg9^ii^	101.5 (3)	Mg2^xxvi^—Mg4—Mg5^x^	65.65 (13)
Mg4—Ru1—Mg9^iii^	101.5 (3)	Mg2^xxvii^—Mg4—Mg5^x^	102.60 (18)
Mg4^i^—Ru1—Mg9^iii^	178.6 (3)	Mg7—Mg4—Mg5^x^	140.70 (12)
Mg9^ii^—Ru1—Mg9^iii^	79.9 (5)	Mg3^xxvi^—Mg4—Mg5^x^	86.21 (13)
Mg4—Ru1—Mg10^iv^	117.56 (12)	Mg3^xxvii^—Mg4—Mg5^x^	158.72 (18)
Mg4^i^—Ru1—Mg10^iv^	117.56 (12)	Ru1—Mg4—Mg5^xi^	67.29 (13)
Mg9^ii^—Ru1—Mg10^iv^	63.03 (17)	Ru3^i^—Mg4—Mg5^xi^	123.54 (15)
Mg9^iii^—Ru1—Mg10^iv^	63.03 (17)	Mg2^xxvi^—Mg4—Mg5^xi^	102.60 (18)
Mg4—Ru1—Mg10^v^	117.56 (12)	Mg2^xxvii^—Mg4—Mg5^xi^	65.65 (13)
Mg4^i^—Ru1—Mg10^v^	117.56 (12)	Mg7—Mg4—Mg5^xi^	140.70 (12)
Mg9^ii^—Ru1—Mg10^v^	63.03 (17)	Mg3^xxvi^—Mg4—Mg5^xi^	158.72 (18)
Mg9^iii^—Ru1—Mg10^v^	63.03 (17)	Mg3^xxvii^—Mg4—Mg5^xi^	86.21 (13)
Mg10^iv^—Ru1—Mg10^v^	107.5 (3)	Mg5^x^—Mg4—Mg5^xi^	75.6 (2)
Mg4—Ru1—Mg6^vi^	69.82 (7)	Ru1—Mg4—Mg6^vi^	59.10 (10)
Mg4^i^—Ru1—Mg6^vi^	69.82 (7)	Ru3^i^—Mg4—Mg6^vi^	115.48 (12)
Mg9^ii^—Ru1—Mg6^vi^	109.77 (10)	Mg2^xxvi^—Mg4—Mg6^vi^	93.39 (10)
Mg9^iii^—Ru1—Mg6^vi^	109.77 (10)	Mg2^xxvii^—Mg4—Mg6^vi^	154.48 (15)
Mg10^iv^—Ru1—Mg6^vi^	62.45 (17)	Mg7—Mg4—Mg6^vi^	85.21 (12)
Mg10^v^—Ru1—Mg6^vi^	169.91 (18)	Mg3^xxvi^—Mg4—Mg6^vi^	57.37 (11)
Mg4—Ru1—Mg6^vii^	69.82 (7)	Mg3^xxvii^—Mg4—Mg6^vi^	142.73 (18)
Mg4^i^—Ru1—Mg6^vii^	69.82 (7)	Mg5^x^—Mg4—Mg6^vi^	58.12 (11)
Mg9^ii^—Ru1—Mg6^vii^	109.77 (10)	Mg5^xi^—Mg4—Mg6^vi^	118.56 (18)
Mg9^iii^—Ru1—Mg6^vii^	109.77 (10)	Ru1—Mg4—Mg6^vii^	59.10 (10)
Mg10^iv^—Ru1—Mg6^vii^	169.91 (18)	Ru3^i^—Mg4—Mg6^vii^	115.48 (12)
Mg10^v^—Ru1—Mg6^vii^	62.45 (17)	Mg2^xxvi^—Mg4—Mg6^vii^	154.48 (15)
Mg6^vi^—Ru1—Mg6^vii^	127.65 (17)	Mg2^xxvii^—Mg4—Mg6^vii^	93.39 (10)
Mg4—Ru1—Mg5^viii^	123.17 (11)	Mg7—Mg4—Mg6^vii^	85.21 (12)
Mg4^i^—Ru1—Mg5^viii^	62.98 (10)	Mg3^xxvi^—Mg4—Mg6^vii^	142.73 (18)
Mg9^ii^—Ru1—Mg5^viii^	55.85 (19)	Mg3^xxvii^—Mg4—Mg6^vii^	57.37 (11)
Mg9^iii^—Ru1—Mg5^viii^	118.1 (2)	Mg5^x^—Mg4—Mg6^vii^	118.56 (18)
Mg10^iv^—Ru1—Mg5^viii^	116.25 (12)	Mg5^xi^—Mg4—Mg6^vii^	58.12 (11)
Mg10^v^—Ru1—Mg5^viii^	59.17 (9)	Mg6^vi^—Mg4—Mg6^vii^	110.25 (18)
Mg6^vi^—Ru1—Mg5^viii^	123.89 (8)	Ru1—Mg4—Mg4^i^	51.46 (9)
Mg6^vii^—Ru1—Mg5^viii^	59.65 (10)	Ru3^i^—Mg4—Mg4^i^	113.42 (10)
Mg4—Ru1—Mg5^ix^	123.17 (11)	Mg2^xxvi^—Mg4—Mg4^i^	146.35 (9)
Mg4^i^—Ru1—Mg5^ix^	62.98 (10)	Mg2^xxvii^—Mg4—Mg4^i^	146.35 (9)
Mg9^ii^—Ru1—Mg5^ix^	55.85 (19)	Mg7—Mg4—Mg4^i^	56.22 (11)
Mg9^iii^—Ru1—Mg5^ix^	118.1 (2)	Mg3^xxvi^—Mg4—Mg4^i^	88.44 (11)
Mg10^iv^—Ru1—Mg5^ix^	59.17 (9)	Mg3^xxvii^—Mg4—Mg4^i^	88.44 (11)
Mg10^v^—Ru1—Mg5^ix^	116.25 (12)	Mg5^x^—Mg4—Mg4^i^	107.37 (12)
Mg6^vi^—Ru1—Mg5^ix^	59.65 (10)	Mg5^xi^—Mg4—Mg4^i^	107.37 (12)
Mg6^vii^—Ru1—Mg5^ix^	123.89 (8)	Mg6^vi^—Mg4—Mg4^i^	58.90 (9)
Mg5^viii^—Ru1—Mg5^ix^	72.62 (18)	Mg6^vii^—Mg4—Mg4^i^	58.90 (9)
Mg4—Ru1—Mg5^x^	62.97 (10)	Ru3^i^—Mg2—Mg2^xxv^	177.23 (9)
Mg4^i^—Ru1—Mg5^x^	123.17 (11)	Ru3^i^—Mg2—Mg3	59.54 (11)
Mg9^ii^—Ru1—Mg5^x^	118.1 (2)	Mg2^xxv^—Mg2—Mg3	123.23 (11)
Mg9^iii^—Ru1—Mg5^x^	55.85 (19)	Ru3^i^—Mg2—Mg4^xxvii^	58.98 (10)
Mg10^iv^—Ru1—Mg5^x^	59.17 (9)	Mg2^xxv^—Mg2—Mg4^xxvii^	121.79 (11)
Mg10^v^—Ru1—Mg5^x^	116.25 (12)	Mg3—Mg2—Mg4^xxvii^	61.51 (10)
Mg6^vi^—Ru1—Mg5^x^	59.65 (10)	Ru3^i^—Mg2—Mg4^xxvi^	58.98 (10)
Mg6^vii^—Ru1—Mg5^x^	123.89 (8)	Mg2^xxv^—Mg2—Mg4^xxvi^	121.79 (11)
Mg5^viii^—Ru1—Mg5^x^	173.20 (15)	Mg3—Mg2—Mg4^xxvi^	61.51 (10)
Mg5^ix^—Ru1—Mg5^x^	106.96 (17)	Mg4^xxvii^—Mg2—Mg4^xxvi^	110.1 (2)
Mg4—Ru1—Mg5^xi^	62.97 (10)	Ru3^i^—Mg2—Mg13^liii^	116.98 (12)
Mg4^i^—Ru1—Mg5^xi^	123.17 (11)	Mg2^xxv^—Mg2—Mg13^liii^	60.87 (8)
Mg9^ii^—Ru1—Mg5^xi^	118.1 (2)	Mg3—Mg2—Mg13^liii^	146.2 (6)
Mg9^iii^—Ru1—Mg5^xi^	55.85 (19)	Mg4^xxvii^—Mg2—Mg13^liii^	149.7 (6)
Mg10^iv^—Ru1—Mg5^xi^	116.25 (12)	Mg4^xxvi^—Mg2—Mg13^liii^	87.3 (5)
Mg10^v^—Ru1—Mg5^xi^	59.17 (9)	Ru3^i^—Mg2—Mg13^liv^	116.98 (12)
Mg6^vi^—Ru1—Mg5^xi^	123.89 (8)	Mg2^xxv^—Mg2—Mg13^liv^	60.87 (8)
Mg6^vii^—Ru1—Mg5^xi^	59.65 (10)	Mg3—Mg2—Mg13^liv^	146.2 (6)
Mg5^viii^—Ru1—Mg5^xi^	106.96 (17)	Mg4^xxvii^—Mg2—Mg13^liv^	87.3 (5)
Mg5^ix^—Ru1—Mg5^xi^	173.20 (15)	Mg4^xxvi^—Mg2—Mg13^liv^	149.7 (6)
Mg5^x^—Ru1—Mg5^xi^	72.62 (18)	Ru3^i^—Mg2—Mg2^xxvi^	57.75 (7)
Mg13^xxii^—Mg1—Mg13	109.471 (6)	Mg2^xxv^—Mg2—Mg2^xxvi^	120.0
Mg13^xxiii^—Mg1—Mg13	109.471 (6)	Mg3—Mg2—Mg2^xxvi^	108.51 (10)
Mg13^ii^—Mg1—Mg13	109.471 (3)	Mg4^xxvii^—Mg2—Mg2^xxvi^	59.07 (9)
Mg11^xxiii^—Mg1—Mg11^xxii^	109.5	Mg4^xxvi^—Mg2—Mg2^xxvi^	107.79 (10)
Mg11^xxiii^—Mg1—Mg11^ii^	109.471 (1)	Mg13^liii^—Mg2—Mg2^xxvi^	92.5 (5)
Mg11^xxii^—Mg1—Mg11^ii^	109.5	Mg13^liv^—Mg2—Mg2^xxvi^	59.26 (8)
Mg11^xxiii^—Mg1—Mg11	109.471 (2)	Ru3^i^—Mg2—Mg2^xxvii^	57.75 (7)
Mg11^xxii^—Mg1—Mg11	109.471 (2)	Mg2^xxv^—Mg2—Mg2^xxvii^	120.0
Mg11^ii^—Mg1—Mg11	109.471 (1)	Mg3—Mg2—Mg2^xxvii^	108.51 (10)
Mg13^xxii^—Mg1—Mg2^xxi^	100.74 (7)	Mg4^xxvii^—Mg2—Mg2^xxvii^	107.79 (10)
Mg13^xxiii^—Mg1—Mg2^xxi^	58.314 (19)	Mg4^xxvi^—Mg2—Mg2^xxvii^	59.07 (10)
Mg13^ii^—Mg1—Mg2^xxi^	58.314 (19)	Mg13^liii^—Mg2—Mg2^xxvii^	59.26 (8)
Mg13—Mg1—Mg2^xxi^	149.79 (7)	Mg13^liv^—Mg2—Mg2^xxvii^	92.5 (5)
Mg11^xxiii^—Mg1—Mg2^xxi^	58.314 (19)	Mg2^xxvi^—Mg2—Mg2^xxvii^	60.0
Mg11^xxii^—Mg1—Mg2^xxi^	100.74 (7)	Ru3^i^—Mg2—Mg11^liii^	116.04 (10)
Mg11^ii^—Mg1—Mg2^xxi^	58.314 (19)	Mg2^xxv^—Mg2—Mg11^liii^	62.38 (8)
Mg11—Mg1—Mg2^xxi^	149.79 (7)	Mg3—Mg2—Mg11^liii^	132.1 (3)
Mg11^xxiii^—Mg1—Mg2^i^	58.314 (19)	Mg4^xxvii^—Mg2—Mg11^liii^	163.2 (3)
Mg11^xxii^—Mg1—Mg2^i^	58.314 (19)	Mg4^xxvi^—Mg2—Mg11^liii^	75.8 (2)
Mg11^ii^—Mg1—Mg2^i^	100.74 (7)	Mg2^xxvi^—Mg2—Mg11^liii^	104.3 (2)
Mg11—Mg1—Mg2^i^	149.79 (7)	Mg2^xxvii^—Mg2—Mg11^liii^	60.88 (9)
Mg2^xxi^—Mg1—Mg2^i^	51.67 (11)	Ru3^i^—Mg2—Mg11^liv^	116.04 (10)
Mg13^xxii^—Mg1—Mg2^xx^	58.314 (19)	Mg2^xxv^—Mg2—Mg11^liv^	62.38 (8)
Mg13^xxiii^—Mg1—Mg2^xx^	100.74 (7)	Mg3—Mg2—Mg11^liv^	132.1 (3)
Mg13^ii^—Mg1—Mg2^xx^	58.314 (19)	Mg4^xxvii^—Mg2—Mg11^liv^	75.8 (2)
Mg13—Mg1—Mg2^xx^	149.79 (7)	Mg4^xxvi^—Mg2—Mg11^liv^	163.2 (3)
Mg11^xxiii^—Mg1—Mg2^xx^	100.74 (7)	Mg2^xxvi^—Mg2—Mg11^liv^	60.88 (9)
Mg11^xxii^—Mg1—Mg2^xx^	58.314 (19)	Mg2^xxvii^—Mg2—Mg11^liv^	104.3 (2)
Mg11^ii^—Mg1—Mg2^xx^	58.314 (19)	Mg11^liii^—Mg2—Mg11^liv^	94.3 (5)
Mg11—Mg1—Mg2^xx^	149.79 (7)	Ru3^i^—Mg2—Mg5^xxviii^	117.44 (9)
Mg2^xxi^—Mg1—Mg2^xx^	51.67 (11)	Mg2^xxv^—Mg2—Mg5^xxviii^	63.81 (7)
Mg2^i^—Mg1—Mg2^xx^	51.67 (11)	Mg3—Mg2—Mg5^xxviii^	84.07 (12)
Mg13^xxii^—Mg1—Mg2^xxiv^	58.314 (19)	Mg4^xxvii^—Mg2—Mg5^xxviii^	142.43 (18)
Mg13^xxiii^—Mg1—Mg2^xxiv^	100.74 (7)	Mg4^xxvi^—Mg2—Mg5^xxviii^	59.11 (9)
Mg13^ii^—Mg1—Mg2^xxiv^	149.79 (7)	Mg13^liii^—Mg2—Mg5^xxviii^	67.7 (5)
Mg13—Mg1—Mg2^xxiv^	58.314 (19)	Mg13^liv^—Mg2—Mg5^xxviii^	120.4 (3)
Mg11^xxiii^—Mg1—Mg2^xxiv^	100.74 (7)	Mg2^xxvi^—Mg2—Mg5^xxviii^	155.63 (11)
Mg11^xxii^—Mg1—Mg2^xxiv^	58.314 (19)	Mg2^xxvii^—Mg2—Mg5^xxviii^	96.55 (10)
Mg11^ii^—Mg1—Mg2^xxiv^	149.79 (7)	Mg11^liii^—Mg2—Mg5^xxviii^	54.3 (2)
Mg11—Mg1—Mg2^xxiv^	58.314 (19)	Mg11^liv^—Mg2—Mg5^xxviii^	125.80 (16)
Mg2^xxi^—Mg1—Mg2^xxiv^	145.86 (9)	Mg12^xvii^—Mg8—Mg12^xviii^	63.5 (4)
Mg2^i^—Mg1—Mg2^xxiv^	94.94 (3)	Mg12^xvii^—Mg8—Ru2	58.51 (14)
Mg2^xx^—Mg1—Mg2^xxiv^	116.61 (4)	Mg12^xviii^—Mg8—Ru2	58.51 (14)
Mg1—Mg11—Mg5^xxxii^	131.6 (2)	Mg12^xvii^—Mg8—Ru2^xxv^	58.51 (14)
Mg1—Mg11—Mg5^xxxiii^	131.6 (2)	Mg12^xviii^—Mg8—Ru2^xxv^	58.51 (14)
Mg5^xxxii^—Mg11—Mg5^xxxiii^	80.7 (4)	Ru2—Mg8—Ru2^xxv^	104.2 (2)
Mg1—Mg11—Mg5^xlv^	131.6 (2)	Mg12^xvii^—Mg8—Mg3^xxv^	101.53 (14)
Mg5^xxxii^—Mg11—Mg5^xlv^	80.7 (4)	Mg12^xviii^—Mg8—Mg3^xxv^	101.53 (14)
Mg5^xxxiii^—Mg11—Mg5^xlv^	80.7 (4)	Ru2—Mg8—Mg3^xxv^	155.7 (3)
Mg1—Mg11—Mg2^viii^	71.9 (3)	Ru2^xxv^—Mg8—Mg3^xxv^	51.49 (9)
Mg5^xxxii^—Mg11—Mg2^viii^	65.57 (12)	Mg12^xvii^—Mg8—Mg3	101.53 (14)
Mg5^xxxiii^—Mg11—Mg2^viii^	144.8 (3)	Mg12^xviii^—Mg8—Mg3	101.53 (14)
Mg5^xlv^—Mg11—Mg2^viii^	102.52 (13)	Ru2—Mg8—Mg3	51.50 (9)
Mg1—Mg11—Mg2^xxx^	71.9 (3)	Ru2^xxv^—Mg8—Mg3	155.7 (3)
Mg5^xxxii^—Mg11—Mg2^xxx^	102.52 (13)	Mg3^xxv^—Mg8—Mg3	152.8 (3)
Mg5^xxxiii^—Mg11—Mg2^xxx^	144.8 (3)	Mg12^xvii^—Mg8—Mg5^xxviii^	157.0 (3)
Mg5^xlv^—Mg11—Mg2^xxx^	65.57 (12)	Mg12^xviii^—Mg8—Mg5^xxviii^	93.47 (18)
Mg2^viii^—Mg11—Mg2^xxx^	58.24 (17)	Ru2—Mg8—Mg5^xxviii^	110.75 (5)
Mg1—Mg11—Mg2^xxix^	71.9 (3)	Ru2^xxv^—Mg8—Mg5^xxviii^	110.75 (5)
Mg5^xxxii^—Mg11—Mg2^xxix^	65.57 (12)	Mg3^xxv^—Mg8—Mg5^xxviii^	82.21 (12)
Mg5^xxxiii^—Mg11—Mg2^xxix^	102.52 (13)	Mg3—Mg8—Mg5^xxviii^	82.21 (12)
Mg5^xlv^—Mg11—Mg2^xxix^	144.8 (3)	Mg12^xvii^—Mg8—Mg5^xxxix^	93.47 (18)
Mg2^viii^—Mg11—Mg2^xxix^	55.23 (17)	Mg12^xviii^—Mg8—Mg5^xxxix^	157.0 (3)
Mg2^xxx^—Mg11—Mg2^xxix^	110.8 (2)	Ru2—Mg8—Mg5^xxxix^	110.75 (5)
Mg1—Mg11—Mg2^xxiv^	71.9 (3)	Ru2^xxv^—Mg8—Mg5^xxxix^	110.75 (5)
Mg5^xxxii^—Mg11—Mg2^xxiv^	102.52 (13)	Mg3^xxv^—Mg8—Mg5^xxxix^	82.21 (12)
Mg5^xxxiii^—Mg11—Mg2^xxiv^	65.57 (12)	Mg3—Mg8—Mg5^xxxix^	82.21 (12)
Mg5^xlv^—Mg11—Mg2^xxiv^	144.8 (3)	Mg5^xxviii^—Mg8—Mg5^xxxix^	109.5 (3)
Mg2^viii^—Mg11—Mg2^xxiv^	110.8 (2)	Mg12^xvii^—Mg8—Mg6^lv^	57.54 (14)
Mg2^xxx^—Mg11—Mg2^xxiv^	143.7 (5)	Mg12^xviii^—Mg8—Mg6^lv^	106.3 (3)
Mg2^xxix^—Mg11—Mg2^xxiv^	58.24 (17)	Ru2—Mg8—Mg6^lv^	113.00 (18)
Mg1—Mg11—Mg2^ix^	71.9 (3)	Ru2^xxv^—Mg8—Mg6^lv^	54.82 (9)
Mg5^xxxii^—Mg11—Mg2^ix^	144.8 (3)	Mg3^xxv^—Mg8—Mg6^lv^	55.95 (7)
Mg5^xxxiii^—Mg11—Mg2^ix^	65.57 (12)	Mg3—Mg8—Mg6^lv^	129.12 (8)
Mg5^xlv^—Mg11—Mg2^ix^	102.52 (13)	Mg5^xxviii^—Mg8—Mg6^lv^	136.12 (17)
Mg2^viii^—Mg11—Mg2^ix^	143.7 (5)	Mg5^xxxix^—Mg8—Mg6^lv^	56.86 (9)
Mg2^xxx^—Mg11—Mg2^ix^	110.8 (2)	Mg12^xvii^—Mg8—Mg6^lvi^	106.3 (3)
Mg2^xxix^—Mg11—Mg2^ix^	110.8 (2)	Mg12^xviii^—Mg8—Mg6^lvi^	57.54 (14)
Mg2^xxiv^—Mg11—Mg2^ix^	55.23 (17)	Ru2—Mg8—Mg6^lvi^	113.00 (18)
Mg1—Mg11—Mg2^xxxi^	71.9 (3)	Ru2^xxv^—Mg8—Mg6^lvi^	54.82 (9)
Mg5^xxxii^—Mg11—Mg2^xxxi^	144.8 (3)	Mg3^xxv^—Mg8—Mg6^lvi^	55.95 (7)
Mg5^xxxiii^—Mg11—Mg2^xxxi^	102.52 (13)	Mg3—Mg8—Mg6^lvi^	129.12 (8)
Mg5^xlv^—Mg11—Mg2^xxxi^	65.57 (12)	Mg5^xxviii^—Mg8—Mg6^lvi^	56.86 (9)
Mg2^viii^—Mg11—Mg2^xxxi^	110.8 (2)	Mg5^xxxix^—Mg8—Mg6^lvi^	136.12 (17)
Mg2^xxx^—Mg11—Mg2^xxxi^	55.23 (17)	Mg6^lv^—Mg8—Mg6^lvi^	101.77 (15)
Mg2^xxix^—Mg11—Mg2^xxxi^	143.7 (5)	Mg12^xvii^—Mg8—Mg6^xiv^	106.3 (3)
Mg2^xxiv^—Mg11—Mg2^xxxi^	110.8 (2)	Mg12^xviii^—Mg8—Mg6^xiv^	57.54 (14)
Mg2^ix^—Mg11—Mg2^xxxi^	58.24 (17)	Ru2—Mg8—Mg6^xiv^	54.82 (9)
Mg1—Mg13—Mg2^viii^	86.3 (6)	Ru2^xxv^—Mg8—Mg6^xiv^	113.00 (18)
Mg1—Mg13—Mg2^xxix^	86.3 (6)	Mg3^xxv^—Mg8—Mg6^xiv^	129.12 (8)
Mg2^viii^—Mg13—Mg2^xxix^	58.26 (16)	Mg3—Mg8—Mg6^xiv^	55.95 (7)
Mg1—Mg13—Mg2^xxiv^	86.3 (6)	Mg5^xxviii^—Mg8—Mg6^xiv^	56.86 (9)
Mg2^viii^—Mg13—Mg2^xxiv^	119.60 (13)	Mg5^xxxix^—Mg8—Mg6^xiv^	136.12 (17)
Mg2^xxix^—Mg13—Mg2^xxiv^	61.47 (16)	Mg6^lv^—Mg8—Mg6^xiv^	162.6 (3)
Mg1—Mg13—Mg2^ix^	86.3 (6)	Mg6^lvi^—Mg8—Mg6^xiv^	75.55 (14)
Mg2^viii^—Mg13—Mg2^ix^	172.5 (12)	Mg12^xvii^—Mg8—Mg6^xv^	57.54 (14)
Mg2^xxix^—Mg13—Mg2^ix^	119.60 (13)	Mg12^xviii^—Mg8—Mg6^xv^	106.3 (3)
Mg2^xxiv^—Mg13—Mg2^ix^	58.26 (16)	Ru2—Mg8—Mg6^xv^	54.82 (9)
Mg1—Mg13—Mg2^xxx^	86.3 (6)	Ru2^xxv^—Mg8—Mg6^xv^	113.00 (18)
Mg2^viii^—Mg13—Mg2^xxx^	61.47 (16)	Mg3^xxv^—Mg8—Mg6^xv^	129.12 (8)
Mg2^xxix^—Mg13—Mg2^xxx^	119.60 (13)	Mg3—Mg8—Mg6^xv^	55.95 (7)
Mg2^xxiv^—Mg13—Mg2^xxx^	172.5 (12)	Mg5^xxviii^—Mg8—Mg6^xv^	136.12 (17)
Mg2^ix^—Mg13—Mg2^xxx^	119.60 (13)	Mg5^xxxix^—Mg8—Mg6^xv^	56.86 (9)
Mg1—Mg13—Mg2^xxxi^	86.3 (6)	Mg6^lv^—Mg8—Mg6^xv^	75.55 (14)
Mg2^viii^—Mg13—Mg2^xxxi^	119.60 (13)	Mg6^lvi^—Mg8—Mg6^xv^	162.6 (3)
Mg2^xxix^—Mg13—Mg2^xxxi^	172.5 (12)	Mg6^xiv^—Mg8—Mg6^xv^	101.77 (15)
Mg2^xxiv^—Mg13—Mg2^xxxi^	119.60 (13)	Ru2^lvii^—Mg12—Ru2^lviii^	110.90 (14)
Mg2^ix^—Mg13—Mg2^xxxi^	61.47 (16)	Ru2^lvii^—Mg12—Ru2^lix^	110.90 (14)
Mg2^xxx^—Mg13—Mg2^xxxi^	58.26 (16)	Ru2^lviii^—Mg12—Ru2^lix^	110.90 (14)
Mg1—Mg13—Mg13^ii^	35.263 (2)	Ru2^lvii^—Mg12—Mg8^lx^	62.89 (4)
Mg2^viii^—Mg13—Mg13^ii^	87.5 (5)	Ru2^lviii^—Mg12—Mg8^lx^	62.89 (4)
Mg2^xxix^—Mg13—Mg13^ii^	116.3 (6)	Ru2^lix^—Mg12—Mg8^lx^	165.5 (3)
Mg2^xxiv^—Mg13—Mg13^ii^	116.3 (6)	Ru2^lvii^—Mg12—Mg8^lxi^	165.5 (3)
Mg2^ix^—Mg13—Mg13^ii^	87.5 (5)	Ru2^lviii^—Mg12—Mg8^lxi^	62.89 (4)
Mg2^xxx^—Mg13—Mg13^ii^	56.3 (6)	Ru2^lix^—Mg12—Mg8^lxi^	62.89 (4)
Mg2^xxxi^—Mg13—Mg13^ii^	56.3 (6)	Mg8^lx^—Mg12—Mg8^lxi^	119.63 (4)
Mg1—Mg13—Mg13^xxii^	35.263 (1)	Ru2^lvii^—Mg12—Mg8^lix^	62.89 (4)
Mg2^viii^—Mg13—Mg13^xxii^	116.3 (6)	Ru2^lviii^—Mg12—Mg8^lix^	165.5 (3)
Mg2^xxix^—Mg13—Mg13^xxii^	87.5 (5)	Ru2^lix^—Mg12—Mg8^lix^	62.89 (4)
Mg2^xxiv^—Mg13—Mg13^xxii^	56.3 (6)	Mg8^lx^—Mg12—Mg8^lix^	119.63 (4)
Mg2^ix^—Mg13—Mg13^xxii^	56.3 (6)	Mg8^lxi^—Mg12—Mg8^lix^	119.63 (4)
Mg2^xxx^—Mg13—Mg13^xxii^	116.3 (6)	Ru2^lvii^—Mg12—Mg6^xliv^	58.45 (9)
Mg2^xxxi^—Mg13—Mg13^xxii^	87.5 (5)	Ru2^lviii^—Mg12—Mg6^xliv^	124.22 (10)
Mg1—Mg13—Mg13^xxiii^	35.263 (2)	Ru2^lix^—Mg12—Mg6^xliv^	124.22 (10)
Mg2^viii^—Mg13—Mg13^xxiii^	56.3 (6)	Mg8^lx^—Mg12—Mg6^xliv^	65.20 (10)
Mg2^xxix^—Mg13—Mg13^xxiii^	56.3 (6)	Mg8^lxi^—Mg12—Mg6^xliv^	136.1 (3)
Mg2^xxiv^—Mg13—Mg13^xxiii^	87.5 (5)	Mg8^lix^—Mg12—Mg6^xliv^	65.20 (10)
Mg2^ix^—Mg13—Mg13^xxiii^	116.3 (6)	Ru2^lvii^—Mg12—Mg6^xliii^	124.22 (10)
Mg2^xxx^—Mg13—Mg13^xxiii^	87.5 (5)	Ru2^lviii^—Mg12—Mg6^xliii^	124.22 (10)
Mg2^xxxi^—Mg13—Mg13^xxiii^	116.3 (6)	Ru2^lix^—Mg12—Mg6^xliii^	58.45 (9)
Mg1—Mg13—Mg5^xxxii^	141.3 (3)	Mg8^lx^—Mg12—Mg6^xliii^	136.1 (3)
Mg2^viii^—Mg13—Mg5^xxxii^	60.3 (3)	Mg8^lxi^—Mg12—Mg6^xliii^	65.20 (10)
Mg2^xxix^—Mg13—Mg5^xxxii^	60.3 (3)	Mg8^lix^—Mg12—Mg6^xliii^	65.20 (10)
Mg2^xxiv^—Mg13—Mg5^xxxii^	93.4 (5)	Mg6^xliv^—Mg12—Mg6^xliii^	82.5 (2)
Mg2^ix^—Mg13—Mg5^xxxii^	125.9 (8)	Ru2^lvii^—Mg12—Mg6^ii^	124.22 (10)
Mg2^xxx^—Mg13—Mg5^xxxii^	93.4 (5)	Ru2^lviii^—Mg12—Mg6^ii^	58.45 (9)
Mg2^xxxi^—Mg13—Mg5^xxxii^	125.9 (8)	Ru2^lix^—Mg12—Mg6^ii^	124.22 (10)
Mg13^ii^—Mg13—Mg5^xxxii^	144.83 (16)	Mg8^lx^—Mg12—Mg6^ii^	65.20 (10)
Mg13^xxii^—Mg13—Mg5^xxxii^	144.83 (16)	Mg8^lxi^—Mg12—Mg6^ii^	65.20 (10)
Mg13^xxiii^—Mg13—Mg5^xxxii^	106.0 (3)	Mg8^lix^—Mg12—Mg6^ii^	136.1 (3)
Mg1—Mg13—Mg5^xxxiii^	141.3 (3)	Mg6^xliv^—Mg12—Mg6^ii^	82.5 (2)
Mg2^viii^—Mg13—Mg5^xxxiii^	125.9 (8)	Mg6^xliii^—Mg12—Mg6^ii^	82.5 (2)
Mg2^xxix^—Mg13—Mg5^xxxiii^	93.4 (5)	Ru2^lvii^—Mg12—Mg12^xlvi^	107.26 (15)
Mg2^xxiv^—Mg13—Mg5^xxxiii^	60.3 (3)	Ru2^lviii^—Mg12—Mg12^xlvi^	58.21 (12)
Mg2^ix^—Mg13—Mg5^xxxiii^	60.3 (3)	Ru2^lix^—Mg12—Mg12^xlvi^	58.21 (12)
Mg2^xxx^—Mg13—Mg5^xxxiii^	125.9 (8)	Mg8^lx^—Mg12—Mg12^xlvi^	109.76 (15)
Mg2^xxxi^—Mg13—Mg5^xxxiii^	93.4 (5)	Mg8^lxi^—Mg12—Mg12^xlvi^	58.24 (18)
Mg13^ii^—Mg13—Mg5^xxxiii^	144.83 (16)	Mg8^lix^—Mg12—Mg12^xlvi^	109.76 (15)
Mg13^xxii^—Mg13—Mg5^xxxiii^	106.0 (3)	Mg6^xliv^—Mg12—Mg12^xlvi^	165.71 (14)
Mg13^xxiii^—Mg13—Mg5^xxxiii^	144.83 (16)	Mg6^xliii^—Mg12—Mg12^xlvi^	108.06 (12)
Mg5^xxxii^—Mg13—Mg5^xxxiii^	65.6 (5)	Mg6^ii^—Mg12—Mg12^xlvi^	108.06 (12)
Ru3^i^—Mg7—Ru3	173.4 (2)	Ru2^lvii^—Mg12—Mg12^xlvii^	58.21 (12)
Ru3^i^—Mg7—Mg4^i^	127.1 (2)	Ru2^lviii^—Mg12—Mg12^xlvii^	107.26 (15)
Ru3—Mg7—Mg4^i^	59.49 (11)	Ru2^lix^—Mg12—Mg12^xlvii^	58.21 (12)
Ru3^i^—Mg7—Mg4	59.50 (11)	Mg8^lx^—Mg12—Mg12^xlvii^	109.76 (15)
Ru3—Mg7—Mg4	127.1 (2)	Mg8^lxi^—Mg12—Mg12^xlvii^	109.76 (15)
Mg4^i^—Mg7—Mg4	67.6 (2)	Mg8^lix^—Mg12—Mg12^xlvii^	58.24 (18)
Ru3^i^—Mg7—Mg3^xx^	122.37 (9)	Mg6^xliv^—Mg12—Mg12^xlvii^	108.06 (12)
Ru3—Mg7—Mg3^xx^	59.46 (8)	Mg6^xliii^—Mg12—Mg12^xlvii^	108.06 (12)
Mg4^i^—Mg7—Mg3^xx^	60.76 (9)	Mg6^ii^—Mg12—Mg12^xlvii^	165.71 (14)
Mg4—Mg7—Mg3^xx^	95.32 (14)	Mg12^xlvi^—Mg12—Mg12^xlvii^	59.998 (1)
Ru3^i^—Mg7—Mg3^xxi^	122.37 (9)	Ru2^lvii^—Mg12—Mg12^xlviii^	58.21 (12)
Ru3—Mg7—Mg3^xxi^	59.46 (8)	Ru2^lviii^—Mg12—Mg12^xlviii^	58.21 (12)
Mg4^i^—Mg7—Mg3^xxi^	60.76 (9)	Ru2^lix^—Mg12—Mg12^xlviii^	107.26 (15)
Mg4—Mg7—Mg3^xxi^	95.32 (14)	Mg8^lx^—Mg12—Mg12^xlviii^	58.24 (18)
Mg3^xx^—Mg7—Mg3^xxi^	109.89 (17)	Mg8^lxi^—Mg12—Mg12^xlviii^	109.76 (15)
Ru3^i^—Mg7—Mg3^xxvi^	59.46 (8)	Mg8^lix^—Mg12—Mg12^xlviii^	109.76 (15)
Ru3—Mg7—Mg3^xxvi^	122.37 (8)	Mg6^xliv^—Mg12—Mg12^xlviii^	108.06 (12)
Mg4^i^—Mg7—Mg3^xxvi^	95.32 (14)	Mg6^xliii^—Mg12—Mg12^xlviii^	165.71 (14)
Mg4—Mg7—Mg3^xxvi^	60.76 (9)	Mg6^ii^—Mg12—Mg12^xlviii^	108.06 (12)
Mg3^xx^—Mg7—Mg3^xxvi^	152.5 (2)	Mg12^xlvi^—Mg12—Mg12^xlviii^	59.998 (1)
Mg3^xxi^—Mg7—Mg3^xxvi^	63.02 (15)	Mg12^xlvii^—Mg12—Mg12^xlviii^	59.998 (1)
Ru3^i^—Mg7—Mg3^xxvii^	59.46 (8)	Mg9^iii^—Mg5—Mg11^iii^	80.5 (3)
Ru3—Mg7—Mg3^xxvii^	122.37 (9)	Mg9^iii^—Mg5—Mg10^ii^	60.5 (3)
Mg4^i^—Mg7—Mg3^xxvii^	95.32 (14)	Mg11^iii^—Mg5—Mg10^ii^	141.1 (3)
Mg4—Mg7—Mg3^xxvii^	60.76 (9)	Mg9^iii^—Mg5—Mg6^xxxv^	104.3 (2)
Mg3^xx^—Mg7—Mg3^xxvii^	63.02 (15)	Mg11^iii^—Mg5—Mg6^xxxv^	138.97 (10)
Mg3^xxi^—Mg7—Mg3^xxvii^	152.5 (2)	Mg10^ii^—Mg5—Mg6^xxxv^	59.41 (15)
Mg3^xxvi^—Mg7—Mg3^xxvii^	109.89 (17)	Mg9^iii^—Mg5—Mg6^xxxiv^	104.3 (2)
Ru3^i^—Mg7—Mg7^xxvi^	57.34 (10)	Mg11^iii^—Mg5—Mg6^xxxiv^	138.97 (10)
Ru3—Mg7—Mg7^xxvi^	117.31 (10)	Mg10^ii^—Mg5—Mg6^xxxiv^	59.41 (15)
Mg4^i^—Mg7—Mg7^xxvi^	149.967 (5)	Mg6^xxxv^—Mg5—Mg6^xxxiv^	80.3 (2)
Mg4—Mg7—Mg7^xxvi^	108.04 (9)	Mg9^iii^—Mg5—Mg4^x^	89.90 (13)
Mg3^xx^—Mg7—Mg7^xxvi^	91.17 (10)	Mg11^iii^—Mg5—Mg4^x^	76.82 (12)
Mg3^xxi^—Mg7—Mg7^xxvi^	147.03 (11)	Mg10^ii^—Mg5—Mg4^x^	101.54 (12)
Mg3^xxvi^—Mg7—Mg7^xxvi^	108.44 (9)	Mg6^xxxv^—Mg5—Mg4^x^	62.62 (11)
Mg3^xxvii^—Mg7—Mg7^xxvi^	59.85 (11)	Mg6^xxxiv^—Mg5—Mg4^x^	142.63 (19)
Ru3^i^—Mg7—Mg7^xxxviii^	57.34 (10)	Mg9^iii^—Mg5—Mg4^xi^	89.90 (13)
Ru3—Mg7—Mg7^xxxviii^	117.31 (10)	Mg11^iii^—Mg5—Mg4^xi^	76.82 (12)
Mg4^i^—Mg7—Mg7^xxxviii^	149.967 (5)	Mg10^ii^—Mg5—Mg4^xi^	101.54 (12)
Mg4—Mg7—Mg7^xxxviii^	108.04 (9)	Mg6^xxxv^—Mg5—Mg4^xi^	142.63 (19)
Mg3^xx^—Mg7—Mg7^xxxviii^	147.03 (11)	Mg6^xxxiv^—Mg5—Mg4^xi^	62.62 (11)
Mg3^xxi^—Mg7—Mg7^xxxviii^	91.17 (10)	Mg4^x^—Mg5—Mg4^xi^	153.3 (2)
Mg3^xxvi^—Mg7—Mg7^xxxviii^	59.85 (11)	Mg9^iii^—Mg5—Mg8^xxxix^	160.6 (3)
Mg3^xxvii^—Mg7—Mg7^xxxviii^	108.44 (9)	Mg11^iii^—Mg5—Mg8^xxxix^	118.9 (3)
Mg7^xxvi^—Mg7—Mg7^xxxviii^	60.000 (1)	Mg10^ii^—Mg5—Mg8^xxxix^	100.1 (2)
Ru3^i^—Mg7—Mg7^xiii^	117.31 (10)	Mg6^xxxv^—Mg5—Mg8^xxxix^	61.79 (16)
Ru3—Mg7—Mg7^xiii^	57.34 (10)	Mg6^xxxiv^—Mg5—Mg8^xxxix^	61.79 (16)
Mg4^i^—Mg7—Mg7^xiii^	108.04 (9)	Mg4^x^—Mg5—Mg8^xxxix^	94.49 (11)
Mg4—Mg7—Mg7^xiii^	149.967 (5)	Mg4^xi^—Mg5—Mg8^xxxix^	94.49 (11)
Mg3^xx^—Mg7—Mg7^xiii^	59.85 (11)	Mg9^iii^—Mg5—Ru1^xi^	53.82 (11)
Mg3^xxi^—Mg7—Mg7^xiii^	108.44 (9)	Mg11^iii^—Mg5—Ru1^xi^	102.8 (2)
Mg3^xxvi^—Mg7—Mg7^xiii^	147.03 (11)	Mg10^ii^—Mg5—Ru1^xi^	55.15 (10)
Mg3^xxvii^—Mg7—Mg7^xiii^	91.17 (10)	Mg6^xxxv^—Mg5—Ru1^xi^	112.88 (16)
Mg7^xxvi^—Mg7—Mg7^xiii^	60.0	Mg6^xxxiv^—Mg5—Ru1^xi^	55.59 (10)
Mg7^xxxviii^—Mg7—Mg7^xiii^	90.0	Mg4^x^—Mg5—Ru1^xi^	142.50 (18)
Ru3^i^—Mg7—Mg7^xii^	117.31 (10)	Mg4^xi^—Mg5—Ru1^xi^	49.73 (9)
Ru3—Mg7—Mg7^xii^	57.34 (10)	Mg8^xxxix^—Mg5—Ru1^xi^	116.72 (13)
Mg4^i^—Mg7—Mg7^xii^	108.04 (9)	Mg9^iii^—Mg5—Ru1^x^	53.82 (11)
Mg4—Mg7—Mg7^xii^	149.967 (5)	Mg11^iii^—Mg5—Ru1^x^	102.8 (2)
Mg3^xx^—Mg7—Mg7^xii^	108.44 (9)	Mg10^ii^—Mg5—Ru1^x^	55.15 (10)
Mg3^xxi^—Mg7—Mg7^xii^	59.85 (11)	Mg6^xxxv^—Mg5—Ru1^x^	55.59 (10)
Mg3^xxvi^—Mg7—Mg7^xii^	91.17 (10)	Mg6^xxxiv^—Mg5—Ru1^x^	112.88 (16)
Mg3^xxvii^—Mg7—Mg7^xii^	147.03 (11)	Mg4^x^—Mg5—Ru1^x^	49.73 (9)
Mg7^xxvi^—Mg7—Mg7^xii^	90.0	Mg4^xi^—Mg5—Ru1^x^	142.50 (18)
Mg7^xxxviii^—Mg7—Mg7^xii^	60.0	Mg8^xxxix^—Mg5—Ru1^x^	116.72 (13)
Mg7^xiii^—Mg7—Mg7^xii^	60.0	Ru1^xi^—Mg5—Ru1^x^	95.43 (15)
Ru2—Mg3—Ru3^i^	167.54 (17)	Mg9^iii^—Mg5—Mg2^xxxix^	130.9 (2)
Ru2—Mg3—Mg2	133.81 (17)	Mg11^iii^—Mg5—Mg2^xxxix^	60.1 (2)
Ru3^i^—Mg3—Mg2	58.66 (11)	Mg10^ii^—Mg5—Mg2^xxxix^	148.93 (15)
Ru2—Mg3—Mg6^xiv^	61.18 (11)	Mg6^xxxv^—Mg5—Mg2^xxxix^	124.6 (2)
Ru3^i^—Mg3—Mg6^xiv^	121.85 (11)	Mg6^xxxiv^—Mg5—Mg2^xxxix^	89.94 (13)
Mg2—Mg3—Mg6^xiv^	98.22 (11)	Mg4^x^—Mg5—Mg2^xxxix^	106.79 (16)
Ru2—Mg3—Mg6^xv^	61.18 (11)	Mg4^xi^—Mg5—Mg2^xxxix^	55.24 (12)
Ru3^i^—Mg3—Mg6^xv^	121.85 (11)	Mg8^xxxix^—Mg5—Mg2^xxxix^	65.62 (15)
Mg2—Mg3—Mg6^xv^	98.22 (11)	Ru1^xi^—Mg5—Mg2^xxxix^	104.94 (7)
Mg6^xiv^—Mg3—Mg6^xv^	113.3 (2)	Ru1^x^—Mg5—Mg2^xxxix^	155.64 (14)
Ru2—Mg3—Mg7^xxxviii^	113.09 (12)	Mg9^iii^—Mg5—Mg2^xxviii^	130.9 (2)
Ru3^i^—Mg3—Mg7^xxxviii^	56.68 (8)	Mg11^iii^—Mg5—Mg2^xxviii^	60.1 (2)
Mg2—Mg3—Mg7^xxxviii^	106.54 (14)	Mg10^ii^—Mg5—Mg2^xxviii^	148.93 (15)
Mg6^xiv^—Mg3—Mg7^xxxviii^	144.32 (16)	Mg6^xxxv^—Mg5—Mg2^xxviii^	89.94 (13)
Mg6^xv^—Mg3—Mg7^xxxviii^	88.45 (13)	Mg6^xxxiv^—Mg5—Mg2^xxviii^	124.6 (2)
Ru2—Mg3—Mg7^xxvi^	113.09 (12)	Mg4^x^—Mg5—Mg2^xxviii^	55.24 (12)
Ru3^i^—Mg3—Mg7^xxvi^	56.68 (8)	Mg4^xi^—Mg5—Mg2^xxviii^	106.79 (16)
Mg2—Mg3—Mg7^xxvi^	106.54 (14)	Mg8^xxxix^—Mg5—Mg2^xxviii^	65.62 (15)
Mg6^xiv^—Mg3—Mg7^xxvi^	88.45 (13)	Ru1^xi^—Mg5—Mg2^xxviii^	155.64 (14)
Mg6^xv^—Mg3—Mg7^xxvi^	144.32 (16)	Ru1^x^—Mg5—Mg2^xxviii^	104.94 (7)
Mg7^xxxviii^—Mg3—Mg7^xxvi^	60.3 (2)	Mg2^xxxix^—Mg5—Mg2^xxviii^	52.39 (15)
Ru2—Mg3—Mg4^xxvi^	125.14 (12)	Ru1^xlv^—Mg9—Ru1^xxxii^	119.34 (7)
Ru3^i^—Mg3—Mg4^xxvi^	58.03 (11)	Ru1^xlv^—Mg9—Ru1^xxxiii^	119.34 (7)
Mg2—Mg3—Mg4^xxvi^	59.54 (11)	Ru1^xxxii^—Mg9—Ru1^xxxiii^	119.34 (7)
Mg6^xiv^—Mg3—Mg4^xxvi^	64.29 (12)	Ru1^xlv^—Mg9—Mg5^xxxii^	70.34 (11)
Mg6^xv^—Mg3—Mg4^xxvi^	155.39 (16)	Ru1^xxxii^—Mg9—Mg5^xxxii^	145.8 (5)
Mg7^xxxviii^—Mg3—Mg4^xxvi^	107.10 (16)	Ru1^xxxiii^—Mg9—Mg5^xxxii^	70.34 (11)
Mg7^xxvi^—Mg3—Mg4^xxvi^	59.10 (13)	Ru1^xlv^—Mg9—Mg5^xlv^	145.8 (5)
Ru2—Mg3—Mg4^xxvii^	125.14 (12)	Ru1^xxxii^—Mg9—Mg5^xlv^	70.34 (11)
Ru3^i^—Mg3—Mg4^xxvii^	58.03 (11)	Ru1^xxxiii^—Mg9—Mg5^xlv^	70.34 (11)
Mg2—Mg3—Mg4^xxvii^	59.54 (11)	Mg5^xxxii^—Mg9—Mg5^xlv^	84.7 (3)
Mg6^xiv^—Mg3—Mg4^xxvii^	155.39 (16)	Ru1^xlv^—Mg9—Mg5^xxxiii^	70.34 (11)
Mg6^xv^—Mg3—Mg4^xxvii^	64.29 (12)	Ru1^xxxii^—Mg9—Mg5^xxxiii^	70.34 (11)
Mg7^xxxviii^—Mg3—Mg4^xxvii^	59.10 (13)	Ru1^xxxiii^—Mg9—Mg5^xxxiii^	145.8 (5)
Mg7^xxvi^—Mg3—Mg4^xxvii^	107.10 (16)	Mg5^xxxii^—Mg9—Mg5^xxxiii^	84.7 (3)
Mg4^xxvi^—Mg3—Mg4^xxvii^	107.0 (2)	Mg5^xlv^—Mg9—Mg5^xxxiii^	84.7 (3)
Ru2—Mg3—Mg3^xii^	54.77 (7)	Ru1^xlv^—Mg9—Mg10^xli^	151.1 (5)
Ru3^i^—Mg3—Mg3^xii^	115.08 (7)	Ru1^xxxii^—Mg9—Mg10^xli^	60.80 (8)
Mg2—Mg3—Mg3^xii^	149.937 (7)	Ru1^xxxiii^—Mg9—Mg10^xli^	60.80 (8)
Mg6^xiv^—Mg3—Mg3^xii^	108.08 (9)	Mg5^xxxii^—Mg9—Mg10^xli^	127.78 (12)
Mg6^xv^—Mg3—Mg3^xii^	58.32 (8)	Mg5^xlv^—Mg9—Mg10^xli^	63.15 (15)
Mg7^xxxviii^—Mg3—Mg3^xii^	58.49 (8)	Mg5^xxxiii^—Mg9—Mg10^xli^	127.78 (12)
Mg7^xxvi^—Mg3—Mg3^xii^	88.83 (10)	Ru1^xlv^—Mg9—Mg10^xlii^	60.80 (8)
Mg4^xxvi^—Mg3—Mg3^xii^	146.21 (10)	Ru1^xxxii^—Mg9—Mg10^xlii^	151.1 (5)
Mg4^xxvii^—Mg3—Mg3^xii^	91.56 (11)	Ru1^xxxiii^—Mg9—Mg10^xlii^	60.80 (8)
Ru2—Mg3—Mg3^xiii^	54.77 (7)	Mg5^xxxii^—Mg9—Mg10^xlii^	63.15 (15)
Ru3^i^—Mg3—Mg3^xiii^	115.08 (7)	Mg5^xlv^—Mg9—Mg10^xlii^	127.78 (12)
Mg2—Mg3—Mg3^xiii^	149.937 (7)	Mg5^xxxiii^—Mg9—Mg10^xlii^	127.78 (12)
Mg6^xiv^—Mg3—Mg3^xiii^	58.32 (8)	Mg10^xli^—Mg9—Mg10^xlii^	104.3 (3)
Mg6^xv^—Mg3—Mg3^xiii^	108.08 (9)	Ru1^xlv^—Mg9—Mg10^xl^	60.80 (8)
Mg7^xxxviii^—Mg3—Mg3^xiii^	88.83 (10)	Ru1^xxxii^—Mg9—Mg10^xl^	60.80 (8)
Mg7^xxvi^—Mg3—Mg3^xiii^	58.49 (8)	Ru1^xxxiii^—Mg9—Mg10^xl^	151.1 (5)
Mg4^xxvi^—Mg3—Mg3^xiii^	91.56 (11)	Mg5^xxxii^—Mg9—Mg10^xl^	127.78 (12)
Mg4^xxvii^—Mg3—Mg3^xiii^	146.21 (10)	Mg5^xlv^—Mg9—Mg10^xl^	127.78 (12)
Mg3^xii^—Mg3—Mg3^xiii^	60.0	Mg5^xxxiii^—Mg9—Mg10^xl^	63.15 (15)
Ru2—Mg3—Mg8	63.44 (18)	Mg10^xli^—Mg9—Mg10^xl^	104.3 (3)
Ru3^i^—Mg3—Mg8	129.0 (2)	Mg10^xlii^—Mg9—Mg10^xl^	104.3 (3)
Mg2—Mg3—Mg8	70.37 (19)	Ru1^xlv^—Mg9—Mg9^xl^	102.8 (2)
Mg6^xiv^—Mg3—Mg8	63.14 (11)	Ru1^xxxii^—Mg9—Mg9^xl^	102.8 (2)
Mg6^xv^—Mg3—Mg8	63.14 (11)	Ru1^xxxiii^—Mg9—Mg9^xl^	50.1 (3)
Mg7^xxxviii^—Mg3—Mg8	149.85 (11)	Mg5^xxxii^—Mg9—Mg9^xl^	106.76 (18)
Mg7^xxvi^—Mg3—Mg8	149.85 (11)	Mg5^xlv^—Mg9—Mg9^xl^	106.76 (18)
Mg4^xxvi^—Mg3—Mg8	97.05 (14)	Mg5^xxxiii^—Mg9—Mg9^xl^	164.2 (2)
Mg4^xxvii^—Mg3—Mg8	97.05 (14)	Mg10^xli^—Mg9—Mg9^xl^	53.24 (18)
Mg3^xii^—Mg3—Mg8	108.65 (15)	Mg10^xlii^—Mg9—Mg9^xl^	53.24 (18)
Mg3^xiii^—Mg3—Mg8	108.65 (15)	Mg10^xl^—Mg9—Mg9^xl^	101.0 (2)
Ru2^xlix^—Mg6—Ru1^vii^	159.90 (16)	Ru1^xlv^—Mg9—Mg9^xli^	50.1 (3)
Ru2^xlix^—Mg6—Mg3^xxxvii^	54.86 (10)	Ru1^xxxii^—Mg9—Mg9^xli^	102.8 (2)
Ru1^vii^—Mg6—Mg3^xxxvii^	108.97 (14)	Ru1^xxxiii^—Mg9—Mg9^xli^	102.8 (2)
Ru2^xlix^—Mg6—Mg3^xxxvi^	54.86 (10)	Mg5^xxxii^—Mg9—Mg9^xli^	106.76 (18)
Ru1^vii^—Mg6—Mg3^xxxvi^	108.97 (14)	Mg5^xlv^—Mg9—Mg9^xli^	164.2 (2)
Mg3^xxxvii^—Mg6—Mg3^xxxvi^	63.35 (17)	Mg5^xxxiii^—Mg9—Mg9^xli^	106.76 (18)
Ru2^xlix^—Mg6—Mg12^ii^	60.57 (15)	Mg10^xli^—Mg9—Mg9^xli^	101.0 (2)
Ru1^vii^—Mg6—Mg12^ii^	139.53 (18)	Mg10^xlii^—Mg9—Mg9^xli^	53.24 (18)
Mg3^xxxvii^—Mg6—Mg12^ii^	105.26 (17)	Mg10^xl^—Mg9—Mg9^xli^	53.24 (18)
Mg3^xxxvi^—Mg6—Mg12^ii^	105.26 (17)	Mg9^xl^—Mg9—Mg9^xli^	59.998 (1)
Ru2^xlix^—Mg6—Mg10^ii^	141.9 (2)	Ru1^xlv^—Mg9—Mg9^xlii^	102.8 (2)
Ru1^vii^—Mg6—Mg10^ii^	58.18 (15)	Ru1^xxxii^—Mg9—Mg9^xlii^	50.1 (3)
Mg3^xxxvii^—Mg6—Mg10^ii^	147.11 (10)	Ru1^xxxiii^—Mg9—Mg9^xlii^	102.8 (2)
Mg3^xxxvi^—Mg6—Mg10^ii^	147.11 (10)	Mg5^xxxii^—Mg9—Mg9^xlii^	164.2 (2)
Mg12^ii^—Mg6—Mg10^ii^	81.4 (2)	Mg5^xlv^—Mg9—Mg9^xlii^	106.76 (18)
Ru2^xlix^—Mg6—Mg5^xxxiv^	120.91 (13)	Mg5^xxxiii^—Mg9—Mg9^xlii^	106.76 (18)
Ru1^vii^—Mg6—Mg5^xxxiv^	64.76 (13)	Mg10^xli^—Mg9—Mg9^xlii^	53.24 (18)
Mg3^xxxvii^—Mg6—Mg5^xxxiv^	147.16 (15)	Mg10^xlii^—Mg9—Mg9^xlii^	101.0 (2)
Mg3^xxxvi^—Mg6—Mg5^xxxiv^	87.45 (12)	Mg10^xl^—Mg9—Mg9^xlii^	53.24 (18)
Mg12^ii^—Mg6—Mg5^xxxiv^	96.34 (13)	Mg9^xl^—Mg9—Mg9^xlii^	59.998 (1)
Mg10^ii^—Mg6—Mg5^xxxiv^	59.68 (12)	Mg9^xli^—Mg9—Mg9^xlii^	59.998 (1)

Symmetry codes: (i) −*x*+1/2, −*y*+1/2, *z*; (ii) −*x*+1, −*y*+1, *z*; (iii) *x*−1/2, *y*−1/2, *z*; (iv) −*x*+1, *y*−1/2, −*z*+3/2; (v) *x*−1/2, −*y*+1, −*z*+3/2; (vi) *x*, −*y*+1/2, −*z*+3/2; (vii) −*x*+1/2, *y*, −*z*+3/2; (viii) −*y*+1/2, −*z*+1, *x*+1/2; (ix) −*z*+1, −*x*+1/2, *y*+1/2; (x) *y*, *z*−1/2, *x*+1/2; (xi) *z*−1/2, *x*, *y*+1/2; (xii) *y*, *z*, *x*; (xiii) *z*, *x*, *y*; (xiv) *y*, *z*−1, *x*; (xv) *z*−1, *x*, *y*; (xvi) *x*, *y*, *z*−1; (xvii) *x*−1, −*y*+1, −*z*+1; (xviii) −*x*+1, *y*−1, −*z*+1; (xix) −*x*+1, −*y*+1, *z*−1; (xx) −*y*+1/2, *z*, −*x*+1/2; (xxi) *z*, −*x*+1/2, −*y*+1/2; (xxii) *x*, −*y*+1, −*z*+1; (xxiii) −*x*+1, *y*, −*z*+1; (xxiv) −*z*+1, *x*+1/2, −*y*+1/2; (xxv) −*x*, −*y*, *z*; (xxvi) *y*, −*z*+1/2, −*x*+1/2; (xxvii) −*z*+1/2, *x*, −*y*+1/2; (xxviii) *x*, −*y*, −*z*+1; (xxix) *y*+1/2, −*z*+1, −*x*+1/2; (xxx) −*x*+1/2, *y*+1/2, −*z*+1; (xxxi) *x*+1/2, −*y*+1/2, −*z*+1; (xxxii) *y*+1/2, *z*, *x*+1/2; (xxxiii) *z*, *x*+1/2, *y*+1/2; (xxxiv) −*z*+1, *x*, −*y*+1; (xxxv) *y*, −*z*+1, −*x*+1; (xxxvi) *z*, *x*, *y*+1; (xxxvii) *y*, *z*, *x*+1; (xxxviii) −*z*+1/2, −*x*+1/2, *y*; (xxxix) −*x*, *y*, −*z*+1; (xl) *x*, −*y*+3/2, −*z*+3/2; (xli) −*x*+3/2, −*y*+3/2, *z*; (xlii) −*x*+3/2, *y*, −*z*+3/2; (xliii) −*y*+1, *z*, −*x*+1; (xliv) *z*, −*x*+1, −*y*+1; (xlv) *x*+1/2, *y*+1/2, *z*; (xlvi) *x*, −*y*+2, −*z*+2; (xlvii) −*x*+2, −*y*+2, *z*; (xlviii) −*x*+2, *y*, −*z*+2; (xlix) *x*, *y*, *z*+1; (l) *y*+1/2, −*z*+3/2, −*x*+1; (li) −*x*+1, *y*+1/2, −*z*+3/2; (lii) −*z*+3/2, −*x*+1, *y*+1/2; (liii) *x*−1/2, −*y*+1/2, −*z*+1; (liv) −*x*+1/2, *y*−1/2, −*z*+1; (lv) −*y*, −*z*+1, *x*; (lvi) −*z*+1, −*x*, *y*; (lvii) *x*+1, −*y*+1, −*z*+1; (lviii) −*x*+1, −*y*+1, *z*+1; (lix) −*x*+1, *y*+1, −*z*+1; (lx) *y*+1, −*z*+1, −*x*+1; (lxi) −*z*+1, −*x*+1, *y*+1.
