# Supplementary material for: Mg44.29Ru7
Source: IUCrdata. 2025 Aug 19;10(Pt 8):x250729. doi: 10.1107/S2414314625007291 (PMC12412686; doi:10.1107/S2414314625007291)
Supplement: Supplementary file 3 [file x-10-x250729-sup4.docx]

**SUPPLEMENTARY MATERIALS:**

**Crystal structure of Mg_44.29_Ru_7_**

**Junhui Li**^a^, **Changzeng Fan**^a,b,^***,Bin Wen**^a^ **and Lifeng Zhang**^c^

^a^ State Key Laboratory of Metastable Materials Science and Technology, Yanshan University,

Qinhuangdao 066004, People’s Republic of China

^b^ Hebei Key Lab for Optimizing Metal Product Technology and Performance, Yanshan University, Qinhuangdao, Hebei 066004, People’s Republic of China

^c^ School of Mechanical and Materials Engineering, North China University of Technology, Beijing, People’s Republic of China

*Correspondence email: [chzfan@ysu.edu.cn](mailto:chzfan@ysu.edu.cn)

The chemical compositions were examined quantitatively by energy dispersive X-ray spectroscopy (EDX) analysis attached to a Hitachi S-3400N SEM for the purpose of guiding the crystal structure refinement. The examined points and areas are designated in Fig. S1, and the corresponding results are listed in Table S1. The deviation relative to the results of refinement of chemical composition is probably caused by the tilt of the single crystal surface to the incident beam. For ease of reading, the atomic ratio of the titled phase was calculated and shown in the last column of Table S1.


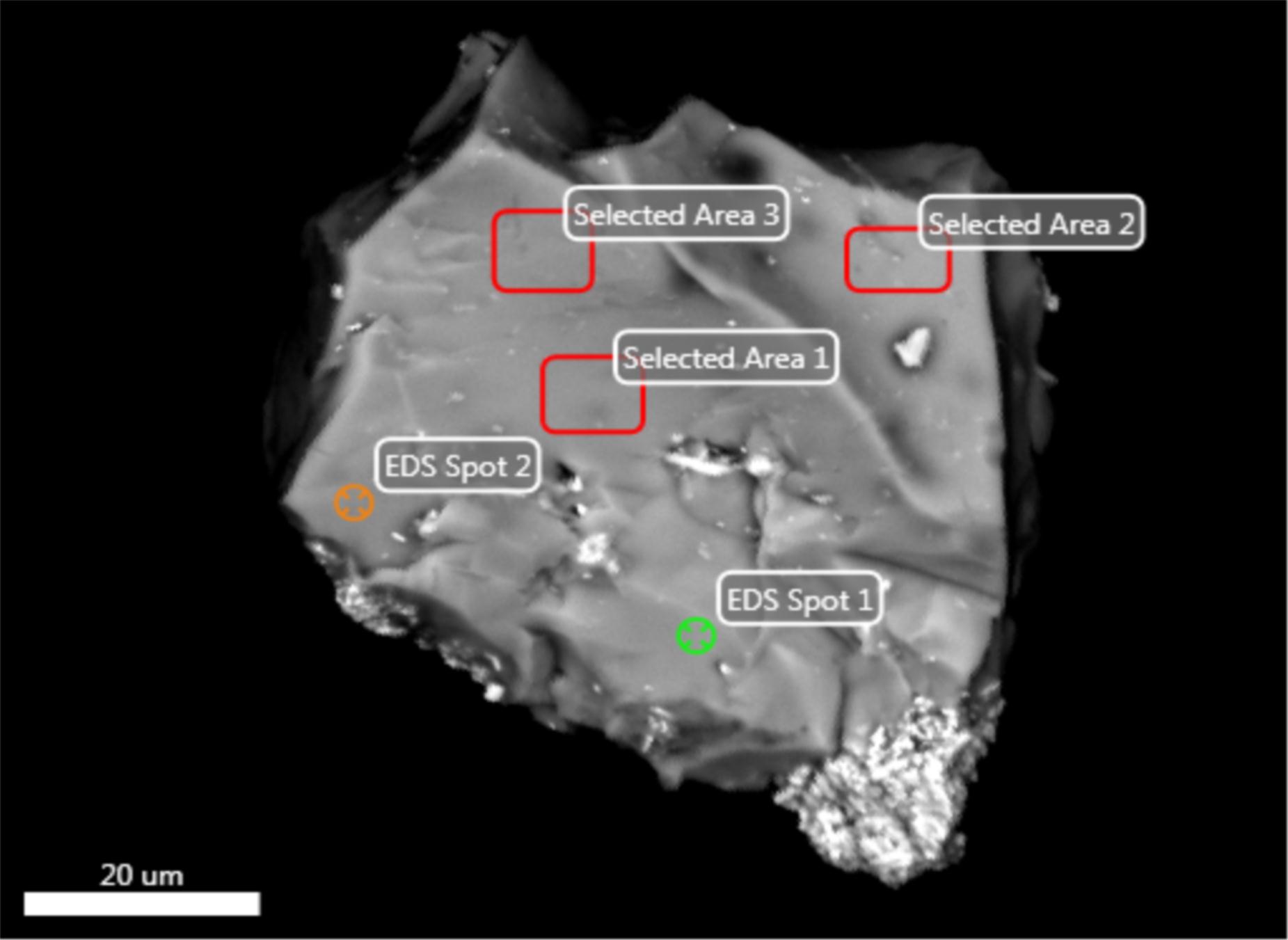


Fig. S1 Single crystal of Mg_44.29_Ru_7_ with selected spots and areas for EDX analysis

**Table S1 EDX results for selected points and areas as designated in Fig. S1**

|  | Element | Weight (%) | Atomic (%) | Error (%) | Mg : Ru |
| --- | --- | --- | --- | --- | --- |
| Area1 | Mg | 63.45 | 87.83 | 4.89 | 7.22:1 |
|  | Ru | 36.55 | 13.17 | 2.13 |  |
| Area2 | Mg | 67.70 | 89.71 | 4.60 | 8.72:1 |
|  | Ru | 32.30 | 10.29 | 2.60 |  |
| Area3 | Mg | 61.55 | 86.93 | 5.03 | 6.65:1 |
|  | Ru | 38.45 | 13.07 | 2.18 |  |
| Spot1 | Mg | 64.16 | 88.15 | 4.89 | 7.43:1 |
|  | Ru | 35.84 | 11.85 | 2.69 |  |
| Spot2 | Mg | 63.80 | 87.99 | 4.71 | 7.32:1 |
|  | Ru | 36.20 | 12.01 | 1.89 |  |
